# Unexpected Latency of *Z*-Stereoretentive Ruthenium Olefin Metathesis Catalysts Bearing Unsymmetrical N-heterocyclic Carbene or Cyclic(alkyl)(amino)carbene Ligands

**DOI:** 10.1021/acs.organomet.2c00428

**Published:** 2022-11-02

**Authors:** Łukasz Grzesiński, Mariusz Milewski, Maryana Nadirova, Anna Kajetanowicz, Karol Grela

**Affiliations:** Biological and Chemical Research Centre, Faculty of Chemistry, University of Warsaw, Żwirki i Wigury Street 101, 02-089 Warsaw, Poland

## Abstract

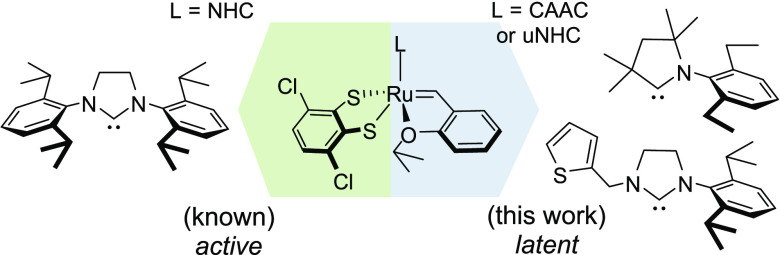

A set of ruthenium complexes bearing a CAAC or uNHC ligand and a dithiocatechol fragment have been obtained and characterized spectroscopically. The activity and *Z*-selectivity of the newly obtained catalysts were studied in selected model CM, self-CM, and RCM olefin metathesis reactions. Intriguingly, and in contrast to structurally related NHC-bearing catalysts **Ru4a** and **Ru4b**, the CAAC and uNHC analogues showed no or only very little activity in olefin metathesis. Interestingly, despite being not productive in metathesis reactions conducted in solution, **Ru8** enabled the synthesis of a model 16-membered macrocyclic lactone of valuable musk smell with excellent chemoselectivity (no C–C double-bond migration was observed) at a concentration 40 times higher than that typically used by organic chemists in similar macrocyclizations (200 mM instead of 5 mM) with excellent *Z*-selectivity. Unfortunately, also here the conversion was low.

## Introduction

**Figure 1:**
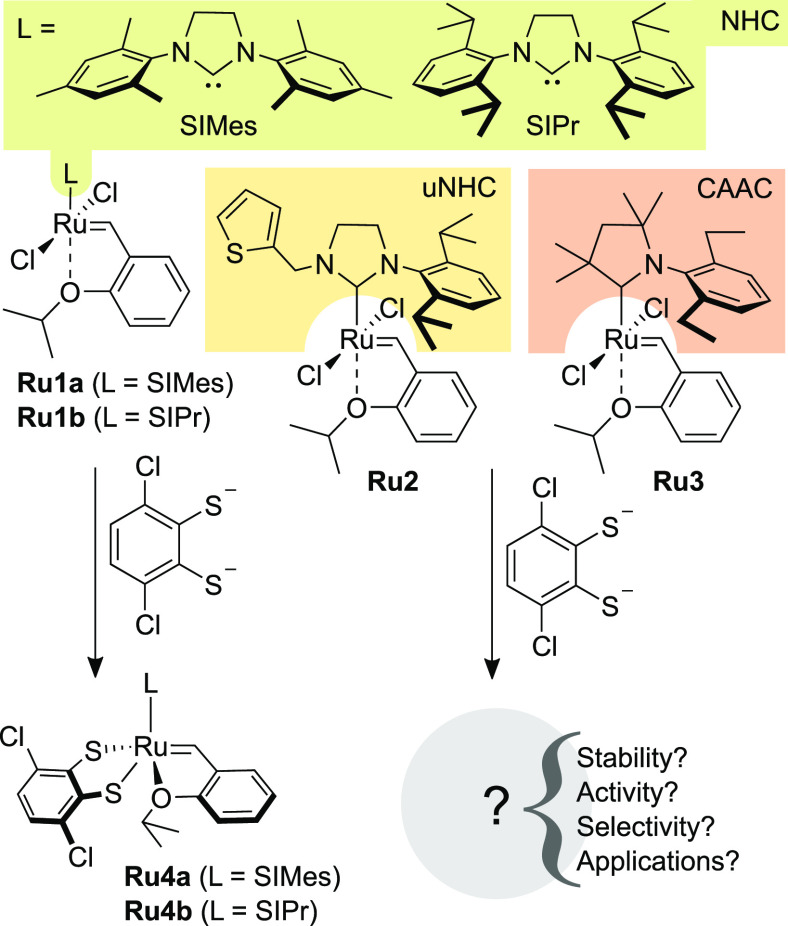
Selected olefin metathesis catalysts **Ru1**–**Ru3** bearing NHC, uNHC, and CAAC ligands and stereoretentive catalysts **Ru4a** and **Ru4b**.

Due to the introduction of well-characterized organometallic complexes based on molybdenum, ruthenium, and tungsten, catalytic olefin metathesis (OM) has become an essential method for the formation of carbon–carbon double bonds, finding myriads of applications.^1−6^ In the case of ruthenium catalysts the introduction of N-heterocyclic carbenes (NHCs) as ligands led to a number of air- and moisture-stable catalysts that were later commercialized and used in an industrial context.^7−9^ In addition to the popular general-purpose NHC ligands, such as SIMes and SIPr (Figure 1), the more specialized unsymmetrical NHC (uNHC)^10−13^ and especially cyclic(alkyl)(amino)carbene (CAAC)^14,15^ ligands have recently been introduced to allow better results in, respectively, industrially important cross-metathesis (CM) of α-olefins^16−20^ and ethenolysis.^19,21^

Another important discovery is related to the development of *stereoretentive* metathesis, where the stereochemistry of the starting material decides the geometry of the product (e.g., *Z*-alkene substrates led to *Z*-alkene products and *E*-alkene substrates led to *E*-alkene products).^22,23^ The first *stereoretentive* catalysts combining a dithiolate anionic ligand and standard SIMes and SIPr neutral NHC ligands were reported by Hoveyda in 2013 (Figure 1).^22^

Therefore, it seems logical to test whether the specialized uNHC and CAAC complexes can, after transformation into the corresponding dithiolate derivatives (Figure 1), act as stereoretentive OM catalysts and what the properties of these systems will be. In the 2018 patent of XiMo Inc., several dithiolate Ru catalysts bearing NHC (e.g., SIMes, SIPr) as well as CAAC ligands have been reported; however, *no single application example* of the catalysts bearing the CAAC ligand was disclosed.^24^ Similarly, in a recent patent from the University of Rennes^25^ revealing some CAAC-dithiocatechol Ru complexes, *only one example* of *Z*-stereoselective CM reaction with the CAAC dithiolate complex was provided.

To the best of our knowledge, the catalytic properties of dithiolate Hoveyda–Grubbs catalysts bearing uNHC or CAAC ligands have not been described in enough detail in the chemical literature; in the present work we intend to fill this gap.

## Results

**Scheme 1:**
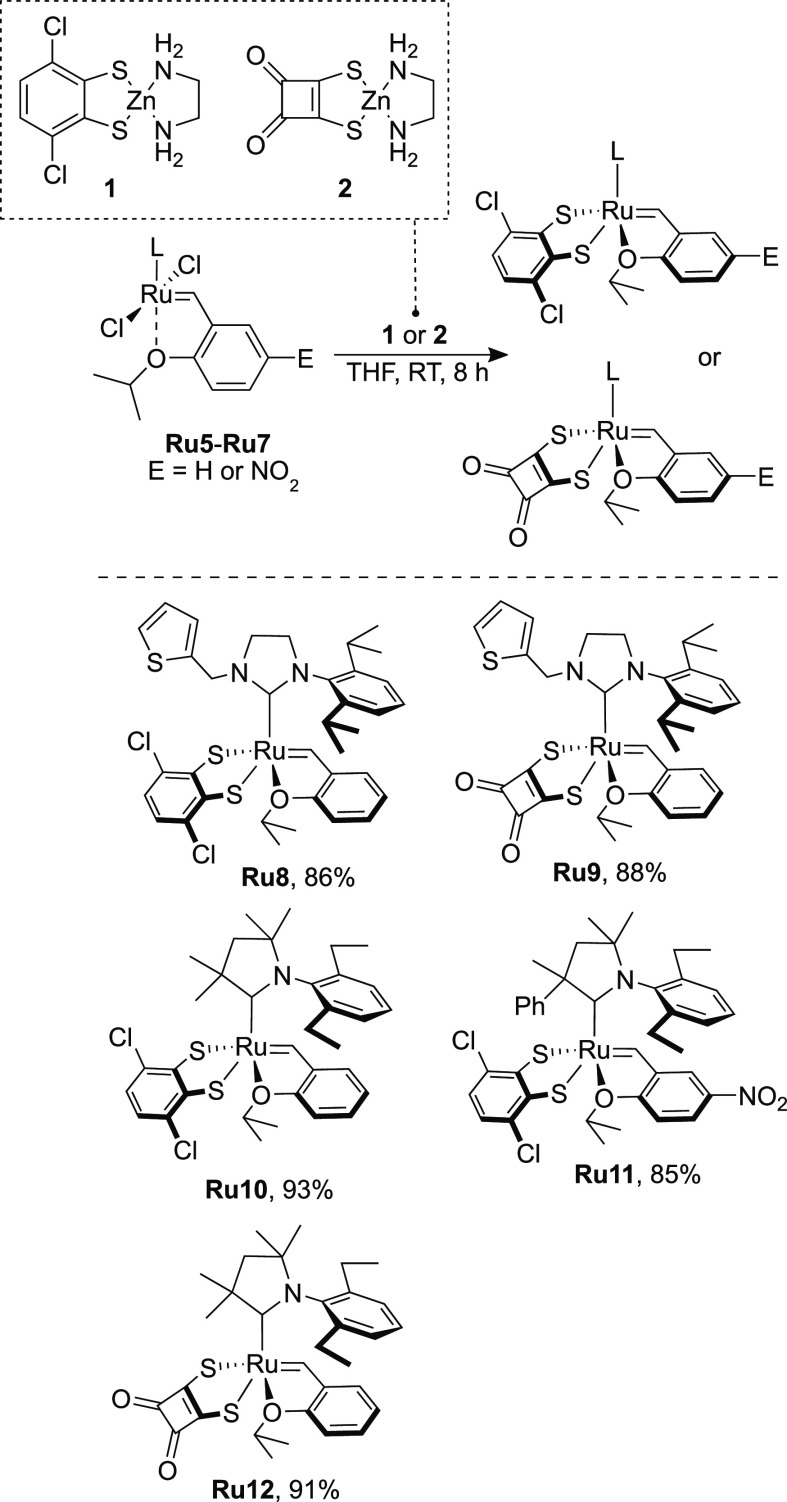
Synthesis of uNHC- and CAAC-Based Ru Complexes Bearing Dithiolate Ligands

Using known or commercially available Ru precursors, such as **Ru5**,^18^**Ru6**,^26^ and **Ru7** (UltraNitroCat),^27^ in reactions with the dithiocatechol zinc salt **1**(28) or squaric acid derivative **2**,^29^ we were able to obtain the five new complexes **Ru8**–**Ru12** (Scheme 1). The synthesis required manipulation in the glovebox. First, zinc dithiolate salts **1** and **2** were prepared from commercially available dithiols and zinc diacetate in the presence of ethylenediamine (for details, see the Supporting Information). Subsequently, the synthesized zinc complexes were reacted with **Ru5**–**Ru7** in THF (RT, 8 h). Filtration through Celite in the glovebox allowed us to obtain the corresponding ruthenium complexes **Ru8**–**Ru12** in excellent yields (Scheme 1). It should be noted that during 1 year of storage in the glovebox, **Ru8**–**Ru10** and **Ru12** maintained their stability according to NMR, while the activated (nitro-substituted)^30^**Ru11** underwent partial decomposition.

**Figure 2:**
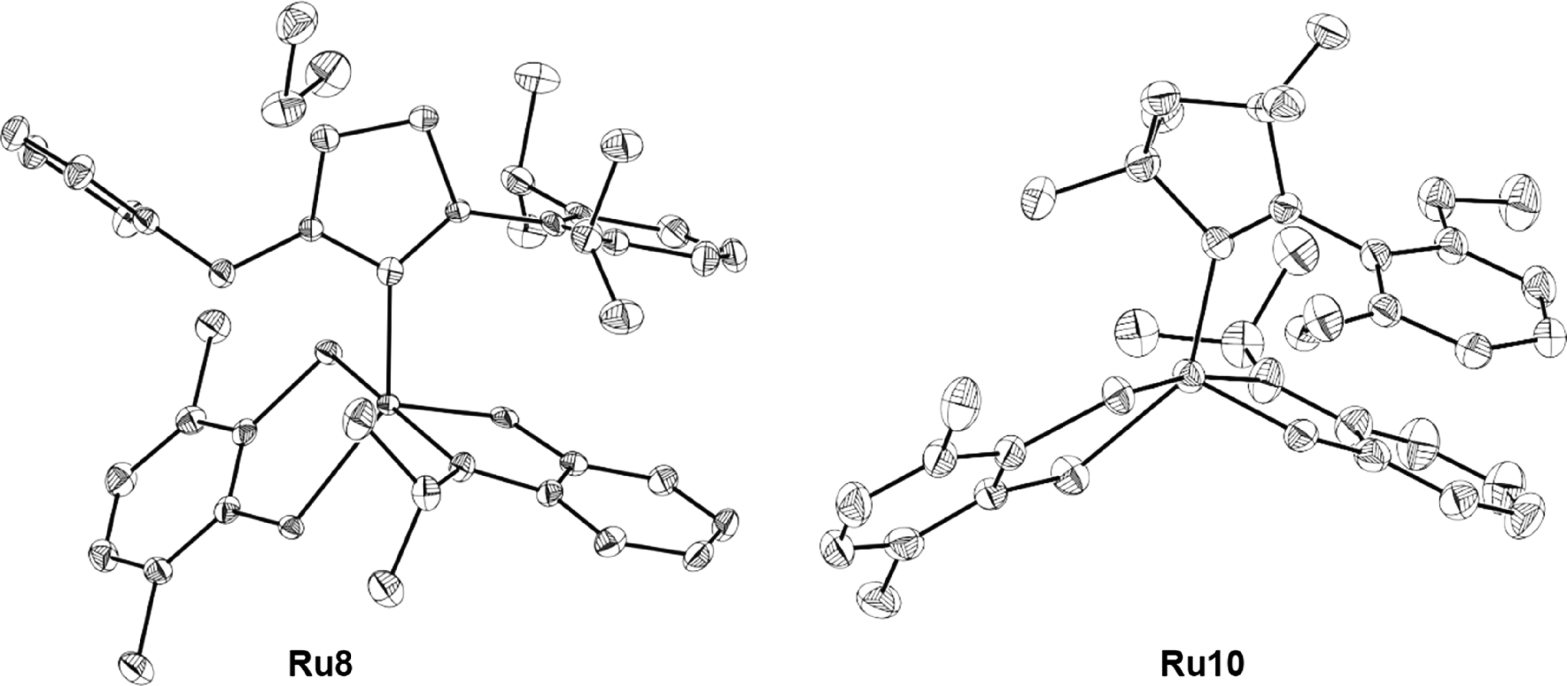
Solid-state molecular structures of **Ru8** and **Ru10** with atom labeling. Displacement ellipsoids are drawn at the 50% probability level. Hydrogen atoms are omitted for clarity.

**Figure 3:**
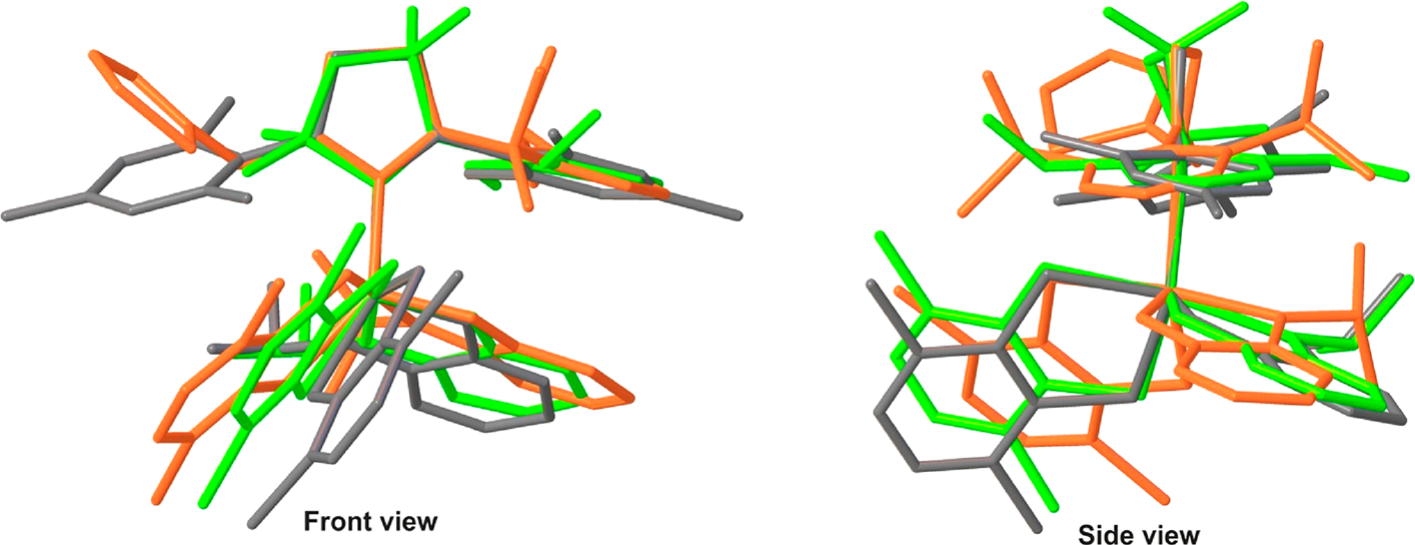
Superimposed molecules of **Ru10** (green), **Ru8** (orange), and **Ru4a** (gray). Hydrogen atoms and molecules of solvents are omitted for clarity.

Crystals of the ruthenium complexes **Ru8** and **Ru10** suitable for diffractometric measurements were obtained *via* liquid-to-liquid diffusion of *n*-heptane into concentrated DCM solutions of catalysts, and their structures were unambiguously confirmed by single-crystal XRD analysis (Figure 2). The investigated compounds crystallize in the monoclinic *P*2_1_/*n* space group with one molecule of the given compound in the asymmetric unit of the crystal lattice. In the case of **Ru8**, the independent part of the unit cell also contains a molecule of dichloromethane. The values of selected parameters describing the geometry of both compounds are reported in Table 1 together with the reference ruthenium complex **Ru4a**, and the superimposition of **Ru8**, **Ru10**, and **Ru4a** depicted in Figure 3 gives a better insight into the structural differences of the studied complexes. The crystallographic data and refinement details as well as a full set of bond lengths and values of valence and torsion angles for **Ru8** and **Ru10** are given in Tables S6–S12 (for details, see the Supporting Information).

**Table 1:** Selected Geometrical Parameters Describing the Molecules **Ru10**, **Ru8**, and **Ru4a**

param (Å, deg)	**Ru10**	**Ru8**	**Ru4a**
Ru–C_(N-heterocycle)_	2.007(4)	2.042(3)	2.071(3)
Ru=C_(benzylidene)_	1.838(4)	1.841(3)	1.830(4)
Ru–O	2.298(3)	2.258(3)	2.319(3)
Ru–S	2.3326(9)/2.2843(9)	2.2909(7)/2.2679(7)	2.2706(10)/2.2954(9)
C_(N-heterocycle)_–Ru–O	103.35(12)	96.84(9)	104.03(12)
C_(N-heterocycle)_–Ru–S	144.39(10)/83.82(10)	139.74(8)/88.68(8)	148.03(11)/85.87(10)
C–Ru–C_(N-heterocycle)_	98.89(15)	101.97(11)	99.91(15)
C–Ru–O	78.70(13)	78.35(10)	78.36(14)
C–Ru–S	116.23(11)/95.56(12)	118.29(8)/94.57(9)	111.69(11)/91.98(13)
O–Ru–S	89.74(7)/171.35(7)	91.41(5)/171.76(5)	87.49(7)/167.13(7)
S–Ru–S	87.00(3)	88.27(2)	88.23(3)

As expected, the Ru atom in both analyzed complexes is pentacoordinated. The values of τ5, which is a parameter characterizing the geometry of coordination centers of this type,^31^ are 0.45 and 0.53 for **Ru10** and **Ru8**, respectively. Therefore, the Ru(II) center, in both cases, adopts a symmetry between a distorted square pyramid and a trigonal bipyramid. Of interest, the change of the carbene ligand in **Ru10** and **Ru8** has an insignificant effect on the length of the Ru–O bond (Table 1) compared to **Ru4a**. The values of this parameter are 2.258(3), 2.298(3), and 2.319(3) Å for **Ru8**, **Ru10**, and **Ru4a**, respectively. The Ru–C_(N-heterocycle)_ bond length for **Ru8** is slightly shorter than for **Ru4a** and significantly shorter for **Ru10** (2.042(3) and 2.071(3) Å, 2.007(4) and 2.071(3) Å, respectively) (Table 1). A comparison of the Ru=C_(benzylidene)_ fragment shows minimal differences in its bond length. In both new complexes, **Ru8** and **Ru10**, this bond is comparable to that observed in the reference complex **Ru4a** (1.841(3), 1.838(4), and 1.830(4) Å, respectively). The noteworthy differences in the presented set of complexes are Ru–S bonds. The lengths of the aforementioned bonds are slightly larger in **Ru10** (2.3326(9) and 2.2843(9) Å) than for **Ru8** and **Ru4a** (2.2909(7), 2.2679(7) Å and 2.2706(10), 2.2954(9) Å, respectively).

In analyzing the geometrical features of the first coordination sphere, special attention should be paid to the O–Ru–S and the C_(NHC)_–Ru–S valence angles, which can be considered as descriptors of the accessibility of the metallic center during the catalytic reaction. Of interest, the values of O–Ru–S1 in **Ru8** and **Ru10** (respectively 171.76(9) and 171.35(2)°) are higher than those within the molecule of the reference complex **Ru4a** (167.13(7)°). In the case of the angle O–Ru–S2 both CAAC complexes have higher values of this parameter than the reference complex, and in comparison with themselves, **Ru8** has the highest angle value (Table 1).

A comparison of the values of the C_(NHC)_–Ru–S1 and C_(NHC)_–Ru–S2 valence angles illustrates interesting structural differences of the complexes. **Ru8** has the lowest value of the C_(NHC)_–Ru–S1 angle (139.74(8)°) and the highest value of the C_(NHC)_–Ru–S2 angle (88.68(8)°) of the entire set. In turn, the values of the aforementioned angles noticed for **Ru10** are in the middle (144.39(10) and 83.82(10)°, respectively).

Interestingly, an analysis of the benzylidene moiety (C–Ru–O angle) shows that this part of the molecules remains unchanged. All presented catalysts have comparable C–Ru–O angle values (Table 1). Comparison of the S–Ru–S angle values reveals some differences related to this moiety. In the case of **Ru10** the value of the above angle is 87.00(3)°, whereas for **Ru8** and **Ru4a** this angle is slightly more obtuse (88.27(2) and 88.23(3)° for **Ru8** and **Ru4a**, respectively).

**Scheme 2:**
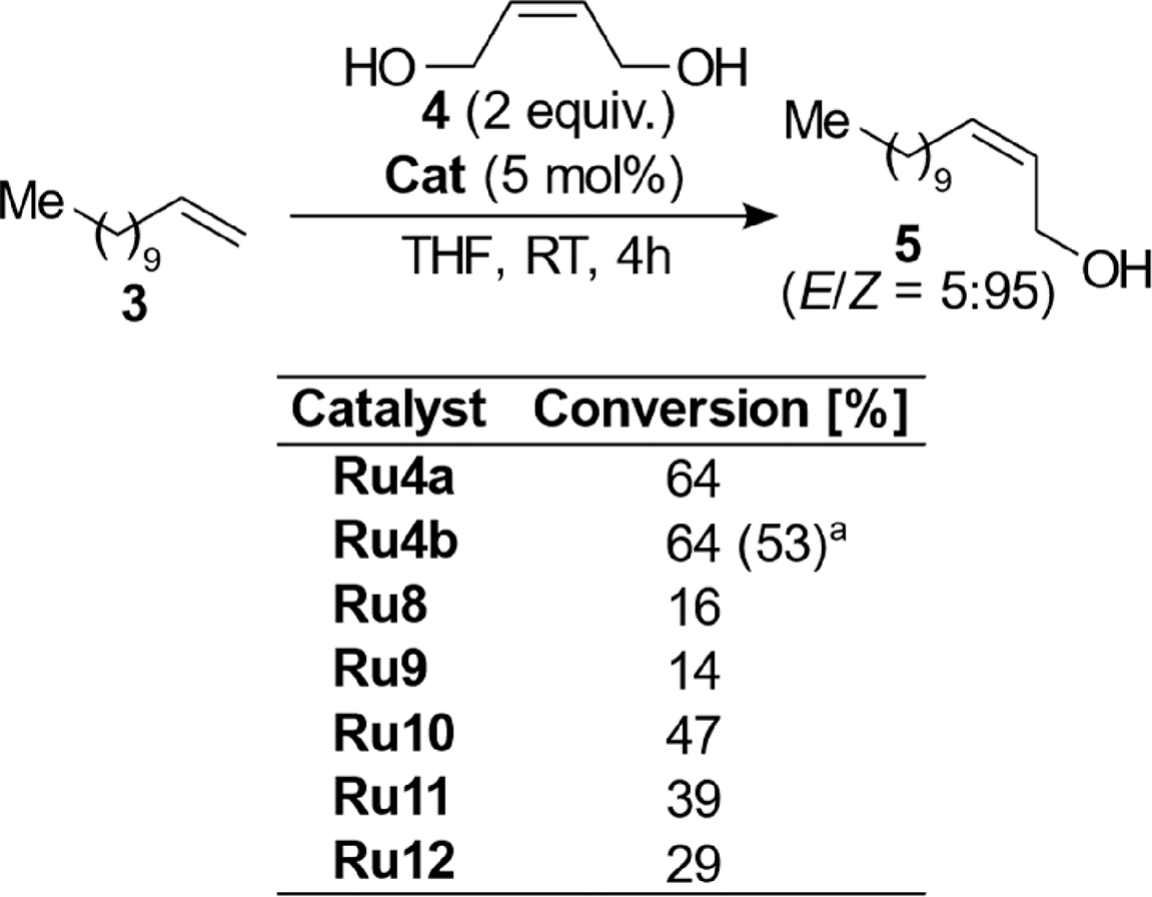
CM Reaction of 1-Dodecene (**3**) and (*Z*)-Butene-1,4-Diol (**4**) Conversion reported in the literature.^32^ Conversions were determined by GC with tetradecane used as an internal standard.

Having a set of new Ru complexes in hand, we opted to test their activity and selectivity in the *Z*-stereoretentive olefin metathesis. To do so, the model CM reaction of 1-dodecene (**3**) and (*Z*)-butene-1,4-diol (**4**) was conducted in THF (RT, 4 h) using known benchmarks **Ru4a** and **Ru4b** and the newly obtained complexes (Scheme 2). We were surprised by the very low activity of all new uNHC- and CAAC-based catalysts, which—with the exception of **Ru10**—gave conversions below 40%. Despite the resulting *E*/*Z* stereoselectivity being very high in all cases (*E*/*Z* = 5/95, which corresponds to the *E*/*Z* ratio of 4/96 reported for **Ru4a** in the literature),^32^ such low chemical activity seems to be disqualifying for practical applications.

**Scheme 3:**
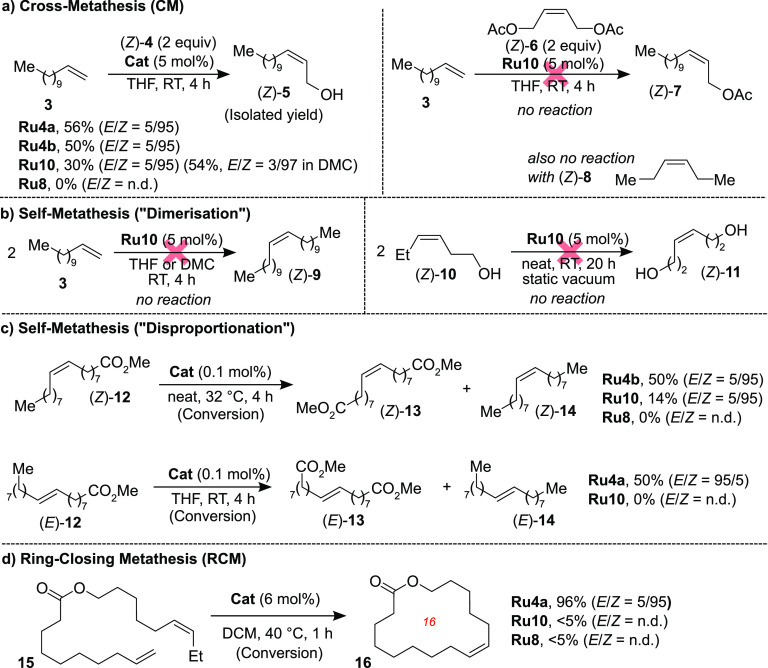
Scope and Limitations of **Ru10** and **Ru8** in Typical Stereoretentive Metathesis Reactions Abbreviations: DCM, dichloromethane; DMC, dimethyl carbonate, THF, tetrahydrofuran.

To take a closer look at the catalytic profile of the most reactive catalyst, **Ru10**, a heterogeneous set of different stereoretentive metathesis reactions have been conducted. First, three typical *Z*-configured cross-metathesis (CM) partners were investigated (Scheme 3a), leading to rather disappointing results. Although **Ru10** has shown some reactivity with (*Z*)-butene-1,4-diol (*Z*)-**4**, leading to product (*Z*)-**5** in 30% isolated yield, this result is still inferior compared to the 50–56% isolated yield obtained with SIMes- and SIPr-based stereoretentive catalysts **Ru4a** and **Ru4b**. Interestingly, **Ru10** gave a slightly higher yield of (*Z*)-**5** in dimethyl carbonate (DMC) compared to THF. Out of curiosity, **Ru8** was also included in some tests. Unfortunately, despite the fact that this uNHC-based catalyst exhibited minimal activity in this CM (see Scheme 2), in a preparative run, we were unable to isolate product (*Z*)-**5** using **Ru8**. Discouragingly, **Ru10** did not enter in a reaction with two other CM partners: (*Z*)-**6** and (*Z*)-**8**. Additionally in the case of self-CM reactions (sometimes termed in the chemical literature as “dimerization” or “homo-metathesis”), shown in Scheme 3b, **Ru10** was impotent. In the case of industrially important self-CM of plain-derived oleic acid methyl ester (*Z*)-**12**,^15^ CAAC-based **Ru10** gave inferior conversion (but equal *Z*-selectivity) compared to the benchmark **Ru4b**, while uNHC-bearing **Ru8** gave no conversion at all (Scheme 3c). In addition, unlike **Ru4a** based on SIMe, **Ru10** gave no reaction with the (*E*-configured) elaidic acid ester (*E*)-**12** (Scheme 3c).

Similarly, in another transformation of commercial value, the RCM macrocyclization of diene **15** leading to musk-smelling (*Z*)-**16**,^33^ both **Ru10** and **Ru8** gave practically no conversion, in contrast to **Ru4a** (Scheme 3d).

**Scheme 4:**
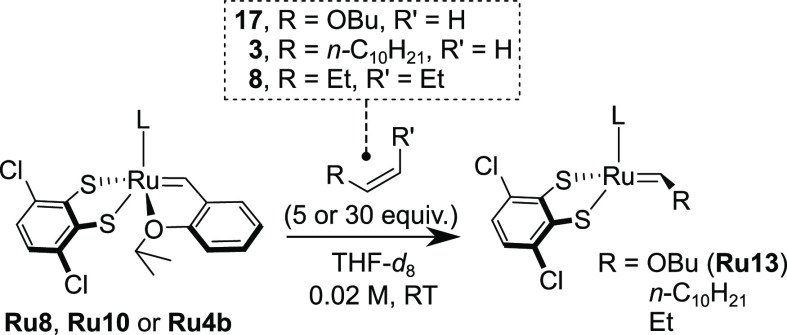
Study of the Initiation of **Ru8** and **Ru10** with Various Olefins For details, see the Supporting Information.

To understand the reasons for such low activities of CAAC- and uNHC-based Ru-dithiocatecholate complexes, the behavior of the catalysts was tested in reactions with *n*-butyl vinyl ether (**17**)—a typical substrate used to study the OM catalyst initiation.^34^ Previously, Grubbs and co-workers assumed that stereoretentive catalysts bearing symmetrical NHC ligands (such as **Ru4a** and **Ru4b**) fully react with a vinyl ether substrate within seconds (<15 s in DCM solution, RT).^35^ In our experiments we decided to use similar conditions but replace DCM with THF-*d*_8_ solvent (Scheme 4). Under these conditions catalyst **Ru4b**, featuring the symmetrical NHC ligand (L = SIPr), gave in our hands full conversion with 30 equiv of *n*-butyl vinyl ether (**17**) in much less than 1 min, which correlates well with the literature results. Interestingly, **Ru8** after 1 min of reaction gave not quite full conversion (79%). Even more interesting is the result of the same reaction conducted for CAAC-substituted catalyst **Ru10**, which after 1 min gave only 3% of the initiation product [Ru]=CH(OBu). After prolonging the reaction to 30 min, no more than 47% of the catalyst reacted with vinyl ether to form the Fisher-type carbene **Ru13**. The experiments with olefin **17** show that the uNHC, and especially the CAAC-based stereoretentive complexes studied herein, *are much more inert* compared to the symmetrical NHC-based catalysts described previously in the literature.

To assess the stability of **Ru8** and **Ru10** during reactions with alkenes typically used in synthetic applications of olefin metathesis, experiments with 1-dodecene (a model terminal olefin) and (*Z*)-3-hexene (a model internal olefin) were conducted (Scheme 4). During the experiment with 5 equiv of a terminal olefin, 1-dodecene (**3**), we observed complete consumption of **Ru8** within 24 h (and no new alkylidene signals were observed in the NMR spectrum), while **Ru10** disappeared slightly more slowly (78% of consumption after 24 h; for details, see the Supporting Information). This behavior was repeated with a model *Z*-configured internal olefin, (*Z*)-3-hexene (**8**, Scheme 4). Although 88% of the initial amount of **Ru8** disappeared during 48 h, in the case of **Ru10**, only 18% of the catalyst was consumed after 2 days in the (*Z*)-3-hexene solution, leaving 82% of the initial catalyst intact. The observed characteristics of the CAAC- and uNHC-based catalysts explain the rather disappointing results of the model reactions presented in Scheme 3. These slowly initiating catalysts **Ru10** and **Ru8** exhibit low activity toward internal olefins and undergo relatively fast decomposition in the presence of olefinic substrates. We suppose that this is related to the formation of a highly unstable active species, which, instead of entering into a productive catalytic cycle and giving the desired product, decomposes quickly, in line with Hoveyda’s observations.^28^

Since the first scientific publication by Villemin,^36^ the manufacturing of macrocyclic unsaturated musk^37^ by RCM has always been conducted under *so-called* high-dilution conditions (around 5 mM diene concentration in the reaction solution). Sadly, this makes this important RCM macrocyclization technology very problematic from an industrial perspective, due to the environmental and economic costs of purchasing, purifying, handling, and then disposing of very large volumes of organic solvents.^38,39^ This pervasive problem in RCM musk production has been partially solved by employing a high-concentration RCM (HC-RCM) based on an *in situ* continuous reactive distillation.^40^ This approach, however, has been limited until now to the nonstereoselective formation of C–C double bonds, thus leading to a musk product as a mixture of geometrical isomers. Because many naturally occurring musks possess *Z*-configured double bonds,^37^ we decided to test whether the studied catalysts can be used to stereoselectively synthesize (*Z*)-macrocycles under HC-RCM conditions. As the target, we selected the 16-membered lactone **16**. Interestingly, this unsaturated macrocycle has been previously obtained in HC-RCM using nonstereoselective catalysts.^40^

**Scheme 5:**
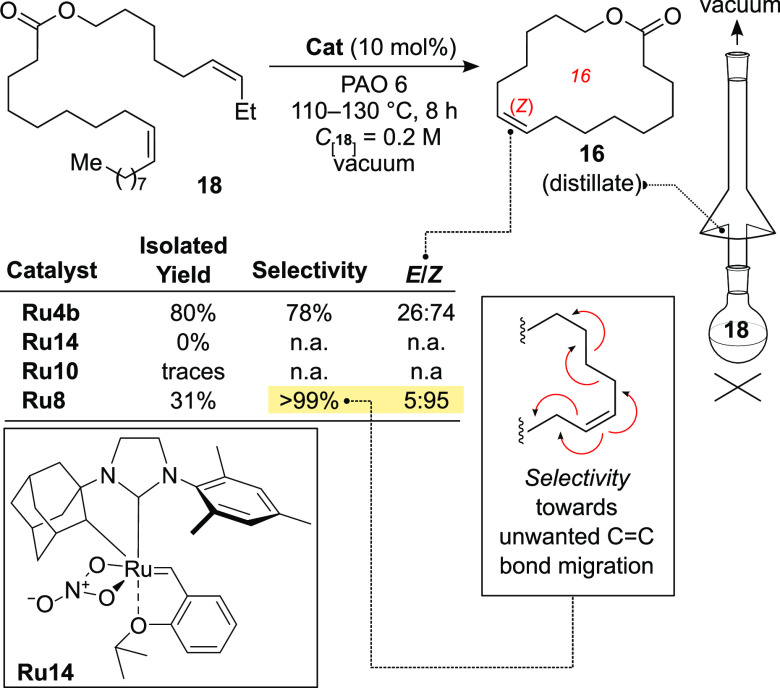
High-Concentration RCM Leading to Stereoselective and Chemoselective Formation of Musk (*Z*)-**16** and Drawing of a Glass Hickmann Adapter Used in Reactive Distillation

Using previously elaborated conditions (PAO-6 engine oil as diluent, 110–130 °C, 8 h, vacuum), we attempted to compare CAAC- and uNHC-based **Ru10** and **Ru8** complexes with known *Z*-stereoretentive (**Ru4b**) and *Z*-stereoselective catalysts (**Ru14**)^34,41,42^ (Scheme 5). While the benchmark complex **Ru4b** provided the desired musk **16** with good isolated yield, the product was obtained as a mixture of *E*- and *Z*-isomers with a rather low selectivity of 26/74. Interestingly, the related **Ru10** did not show any trace of the product under the same conditions. The same result was obtained in the case of cyclometalated **Ru14**—a very selective and useful catalyst in solution—which, unfortunately, under much harsher conditions of HC-RCM underwent decomposition. Finally, we were pleased to see that in the presence of **Ru8** musk lactone **16** can be obtained at 200 mM concentration with high *Z*-selectivity (*E*/*Z* = 5/95), although in a moderate isolated yield of 31%. Importantly, and in contrast to **Ru4b**, the parasitic C–C double-bond migration was practically not occurring in this case (>99% of selectivity).

## Conclusion

A set of ruthenium complexes bearing a CAAC or uNHC ligand and a dithiocatechol fragment have been obtained and characterized spectroscopically. Their activity and selectivity were studied in selected model *Z*-stereoretentive olefin metathesis reactions, such as CM, self-CM, and RCM. Interestingly, and in visible contrast to structurally related NHC-bearing catalysts **Ru4a** and **Ru4b**, these complexes showed no or only very limited activity in olefin metathesis. The solid-state geometry of two representative CAAC and uNHC complexes and their reactions with model olefins were studied in the hope of shedding some light on the intriguing low reactivity of these systems. Interestingly, despite being unproductive in metathesis reactions conducted in solution, **Ru8** allowed us to obtain a valuable 16-membered macrocyclic lactone possessing a musk smell with excellent chemoselectivity (no C–C double bond migration!) at concentrations *40 times higher* than those typically used by organic chemists in similar macrocyclizations (200 mM instead of 5 mM) and with excellent *Z*-selectivity. To our knowledge, this is the second example of an HC-RCM process published in the literature, and the first one in which a musk is formed fully stereoselectively as the *Z*-isomer. Unfortunately, the relatively low yield of this process should be considered a serious limitation. Further research to obtain geometrically defined musk macrocycles *via* stereoselective reactive distillation will be continued and published in due time.

## Supporting Information Available

Experimental details, crystal data for **Ru8** and **Ru10**, and NMR spectra of new complexes (PDF)
