# Supplementary material for: Unexpected Latency of Z-Stereoretentive Ruthenium Olefin Metathesis Catalysts Bearing Unsymmetrical N-heterocyclic Carbene or Cyclic(alkyl)(amino)carbene Ligands
Source: Organometallics. 2022 Nov 2;42(18):2453–9. doi: 10.1021/acs.organomet.2c00428 (PMC10526643; doi:10.1021/acs.organomet.2c00428)
Supplement: Supplementary file 1 — om2c00428_si_001.pdf [file om2c00428_si_001.pdf]

SUPPORTING INFORMATION

On the Unexpected Latency of Z-Stereoretentive Ruthenium Olefin Metathesis Catalysts Bearing Unsymmetrical N-Heterocyclic Carbene or Cyclic(Alkyl)(Amino)Carbene Ligands

Łukasz Grzesiński,[a‡] Mariusz Milewski,[a‡] Maryana Nadirova,[a‡] Anna Kajetanowicz,[a,*] and Karol Grela[a,*]

[a] Biological and Chemical Research Centre, Faculty of Chemistry, University of Warsaw, Żwirki i Wigury 101, 02-089 Warsaw, Poland

email: a.kajetanowicz@uw.edu.pl, prof.grela@gmail.com

General Remarks ... 3
Reagents and Solvents ... 3
General Procedure for Synthesis of Ru Complexes ... 4
Synthesis of ruthenium complex **Ru8** ... 4
Synthesis of ruthenium complex **Ru9** ... 5
Synthesis of ruthenium complex **Ru10** ... 5
Synthesis of ruthenium complex **Ru11** ... 6
Synthesis of ruthenium complex **Ru12** ... 6
Cross-Metathesis Reactions ... 8
Cross-Metathesis reaction between 1-dodecene and (*Z*)-2-butene-1,4-diol—optimization ... 8
Cross-Metathesis reaction of 1-dodecene and (*Z*)-2-butene-1,4-diol in DMC ... 8
Preparation of stock solutions in DMC ... 8
Procedure of the CM reaction of 1-dodecene and butene-1,4-diol in DMC ... 9
Cross metathesis reaction of 1-dodecene and (*Z*)-2-butene-1,4-diol in THF ... 9
Preparation of stock solutions in THF ... 9
Procedure of the CM reaction of 1-dodecene and butene-1,4-diol in THF ... 10
Cross-Metathesis reaction between 1-dodecene and (*Z*)-1,4-diacetoxy-2-butene or (*Z*)-3-hexene . 10
Self-Metathesis—Dimerization of 1-dodecene ... 10
Self-Metathesis—Dimerization of hex-3-ene-1-ol ... 11
Self-Metathesis—Disproportionation of oleic acid methyl ester ... 11
Self-Metathesis—Disproportionation of elaidic acid methyl ester ... 11
Ring-Closing Metathesis ... 11
Ring-Closing Metathesis at high concentration ... 12
Activity/stability test ... 13
Copies of NMR Spectra ... 18
Crystallographic Information ... 23
References ... 29

General Remarks

All reactions requiring exclusion of oxygen and moisture were carried out in dry glassware with dry solvents (SPS MBraun) under a dry and oxygen free argon atmosphere using standard Schlenk technique. The addition of dry solvents or reagents was carried out using argon flushed stainless steel cannulas and plastic syringes.

For spectroscopic and analytic characterizations the following devices were used:

Analytical thin layer chromatography (TLC) was performed on Merck Silica gel 60 F_{254} precoated aluminum sheets. Components were visualized by observation under UV light (254 nm or 365 nm) or dyed by aqueous $KMnO_4$ or anisaldehyde reagent.

Flash column chromatography was carried out using silica gel 60 (230 – 400 mesh), purchased from Merck.

GC chromatograms were recorded using a PerkinElmer Clarus 580 model. As capillary column, an IntertCap 5MS-Sil column was employed with helium as carrier gas. GC conversions were determined based on the ratio of an internal standard (durene or tetradecane) and the starting material.

^{1}H NMR spectra were recorded in dichloromethane-d_2 at room temperature on Agilent Mercury spectrometers (400 MHz). The data were interpreted in first order spectra. Chemical shifts δ are reported in parts per million (ppm) downfield from trimethylsilane as reference to residual solvent signal: dichloromethane-d_2 [δ_H = 5.32 ppm]. The following abbreviations are used to indicate the signal multiplicity: s (singlet), d (doublet), t (triplet), q (quartet), quin (quintet), sext (sextet), dd (doublet of doublet), dt (doublet of triplet), ddd (doublet of doublet of doublet), etc., br. s (broad signal), m (multiplet). Coupling constants (*J*) are given in Hz and refer to H,H-couplings.

^{13}C NMR spectra were recorded at room temperature on Agilent Mercury 400 MHz spectrometers. The spectra were recorded in dichloromethane-d_2. Chemical shifts are reported in δ units relative to the solvent signal: dichloromethane-d_2 [δ_C = 53.84 ppm (central line of the quintet)]. If no coupling constants are given, the multiplicity refers to ^{1}H-decoupled spectra, otherwise the coupling constants belong to heteroatoms.

High resolution mass spectra (HR-MS) High resolution mass spectroscopy was obtained on AutoSpec Premier spectrometer.

Elemental Analyses were carried out at the Polish Academy of Science, Institute of Organic Chemistry.

IR spectra were recorded on a Perkin-Elmer Spectrum One FTIR spectrometer. Substances were applied as a film, solid or in solution. The obtained data was processed with the software Omni32. Wavenumbers are given in cm^{-1}.

Reagents and Solvents

All reagents were purchased from Sigma-Aldrich, Apeiron Synthesis and POCH and used without

further purification unless stated otherwise.

General Procedure for Synthesis of Ru Complexes

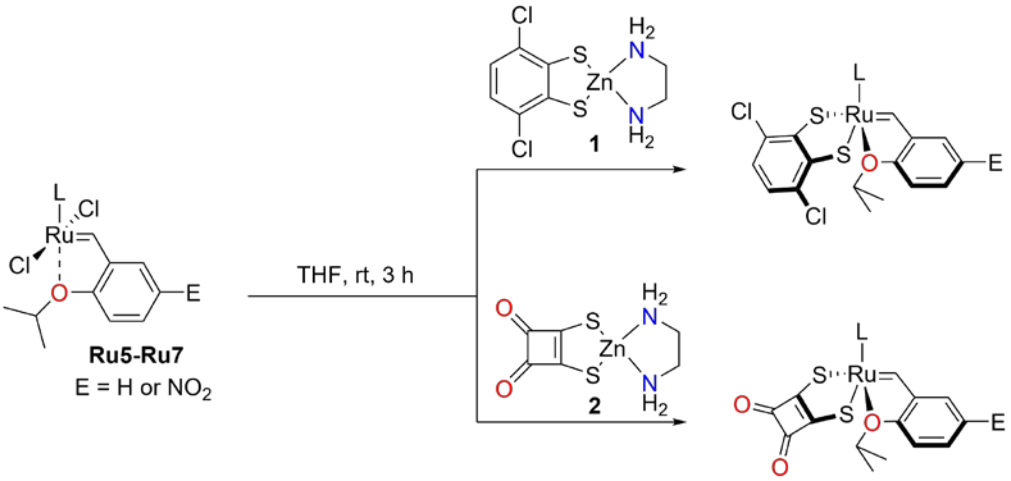

In a glovebox a 20 mL vial was charged with corresponding ruthenium complex (1 equiv.) dissolved in anhydrous THF followed by zinc salt (2.0 equiv.). The resulting mixture was stirred for 3 hours at room temperature. After the reaction time was completed, THF was evaporated, the dark brown residue was dissolved in DCM (10 mL), and the obtained solution was filtered through celite. Then, DCM was evaporated to dryness, and residue was crystalized from DCM/hexane, the precipitate was filtered, and dried in vacuum.

Synthesis of ruthenium complex Ru8

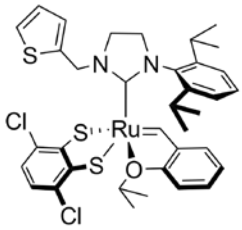

Following the general procedure, using **Ru5** (200 mg, 0.31 mmol, 1 equiv.), dichlorodithiocatechol complex **1** (207 mg, 0.62 mmol, 2.0 equiv.), and THF (15 mL). The desired product was obtained as a brown solid (210 mg, 0.27 mmol, 86% yield).

^{1}H NMR (400 MHz, CD_2Cl_2) δ 14.27 (s, 1H), 7.37 – 7.28 (m, 3H), 7.16 (t, *J* = 7.7 Hz, 1H), 7.08 (dd, *J* = 3.5, 1.2 Hz, 1H), 7.01 (d, *J* = 8.5 Hz, 1H), 7.00 – 6.95 (m, 2H), 6.89 (d, *J* = 8.1 Hz, 1H), 6.77 (td, *J* = 7.5, 0.8 Hz, 1H), 6.67 (dd, *J* = 7.5, 1.7 Hz, 1H), 6.53 (dd, *J* = 7.8, 1.5 Hz, 1H), 5.22 (hept, *J* = 6.3 Hz, 1H), 4.98 (d, *J* = 14.9 Hz, 1H), 4.81 – 4.73 (m, 1H), 4.02 – 3.78 (m, 2H), 3.75 – 3.57 (m, 2H), 3.39 (p, *J* = 6.7 Hz, 1H), 2.45 (h, *J* = 6.8 Hz, 1H), 1.84 (dd, *J* = 10.8, 6.3 Hz, 9H), 1.24 (d, *J* = 6.8 Hz, 3H), 0.97 (d, *J* = 6.8 Hz, 3H), 0.55 (d, *J* = 6.8 Hz, 3H).

^{13}C NMR (101 MHz, CD_2Cl_2) δ 254.2, 254.0, 218.0, 156.6, 154.3, 148.3, 146.4, 143.1, 140.4, 138.3, 137.3, 132.2, 130.6, 129.7, 128.7, 128.4, 127.2, 127.1, 124.6, 124.3, 123.9, 123.8, 122.8, 122.4, 112.6, 76.1, 54.0, 52.4, 48.6, 29.0, 28.5, 27.7, 26.8, 24.8, 24.6, 22.1, 21.7.

IR $\tilde{\nu}$: 3068, 2961, 2929, 2868, 1589, 1574, 1544, 1524, 1474, 1442, 1414, 1394, 1385, 1375, 1364, 1331, 1306, 1252, 1208, 1156, 1138, 1110, 1094, 1062, 934, 816, 806, 784, 758, 740, 732, 701, 618, 599, 567, 557,

484, 470, 452, 3068, 2961, 2929, 2868, 1589, 1574, 1544, 1524, 1474, 1442, 1414, 1394, 1385, 1375, 1364, 1331, 1306, 1252, 1208, 1156, 1138, 1110, 1094, 1062, 934, 816, 806, 784, 758, 740, 732, 701, 618, 599, 567, 557, 484, 470, 452 cm^{-1}.

HRMS (ESI TOF *m/z*) calculated for $C_{36}H_{40}Cl_2N_2ORuS_3$ $[M]^+$: 784.0723, Found: 784.0711.

EA: calculated for $C_{36}H_{40}Cl_2N_2ORuS_3$ + 0.25×CH_2Cl_2: C: 52.92; H: 4.89; Found C: 52.63; H: 4.72.

Synthesis of ruthenium complex Ru9

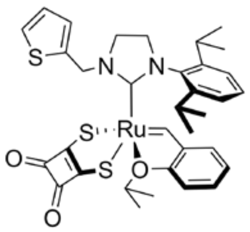

Following the general procedure, using **Ru5** (200 mg, 0.31 mmol, 1 equiv.), squaric acid derivative **2** (125 mg, 0.46 mmol, 1.5 equiv.), and THF (15 mL). The desired product was obtained as a brown solid (195 mg, 0.27 mmol, 88% yield).

^{1}H NMR (400 MHz, CD_2Cl_2) δ 15.39 (s, 1H), 7.51 – 7.38 (m, 2H), 7.33 (dd, *J* = 7.9, 1.5 Hz, 1H), 7.23 – 7.13 (m, 2H), 7.07 – 6.99 (m, 2H), 6.87 (t, *J* = 7.4 Hz, 1H), 6.81 (dd, *J* = 7.7, 1.7 Hz, 1H), 6.52 (dd, *J* = 7.8, 1.5 Hz, 1H), 5.16 (dd, *J* = 98.3, 14.8 Hz, 2H), 5.15 (p, *J* = 6.2 Hz, 1H), 3.98 – 3.77 (m, 2H), 3.76 – 3.66 (m, 2H), 3.16 (p, *J* = 6.8 Hz, 1H), 2.33 (p, *J* = 6.8 Hz, 1H), 1.76 (dd, *J* = 10.3, 6.3 Hz, 9H), 1.18 (d, *J* = 6.8 Hz, 3H), 0.95 (d, *J* = 6.7 Hz, 3H), 0.55 (d, *J* = 6.8 Hz, 3H).

^{13}C NMR (101 MHz, CD_2Cl_2) δ 268.8, 225.0, 214.3, 209.1, 188.0, 186.8, 156.9, 147.8, 146.6, 140.5, 137.4, 136.2, 130.6, 130.3, 129.1, 127.6, 127.4, 124.7, 124.2, 123.3, 113.0, 77.0, 54.5, 54.5, 54.4, 54.3, 54.3, 54.0, 53.7, 53.5, 51.9, 48.7, 28.9, 28.5, 27.6, 26.8, 24.5, 24.1, 22.1, 21.2.

IR $\tilde{\nu}$: 3064, 2973, 2934, 2876, 1806, 1738, 1712, 1646, 1588, 1575, 1524, 1452, 1422, 1387, 1370, 1332, 1297, 1276, 1237, 1194, 1152, 1128, 1111, 1091, 1062, 1035, 988, 922, 875, 810, 780, 741, 706, 566, 520 cm^{-1}.

HRMS (ESI TOF *m/z*) calculated for $C_{34}H_{39}N_2O_3RuS_3$ $[M+H]^+$: 721.1166, Found: 721.1157.

EA: calculated for $C_{34}H_{38}N_2O_3RuS_3$ + 0.25×CH_2Cl_2: C: 55.50; H: 5.24; Found C: 55.45; H: 5.44.

Synthesis of ruthenium complex Ru10

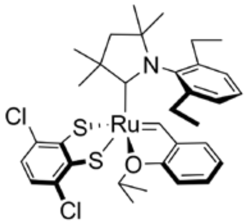

Following the general procedure, using **Ru6** (200 mg, 0.35 mmol, 1 equiv.), zinc dichlorodithiocatechol complex **1** (232 mg, 0.70 mmol, 2.0 equiv.), and THF (15 mL). The desired product was obtained as a brown solid (230 mg, 0.32 mmol, 93% yield).

^{1}H NMR (400 MHz, CD_2Cl_2) δ 13.88 (s, 1H), 7.43 (dt, *J* = 7.6, 1.2 Hz, 1H), 7.38 – 7.28 (m, 1H), 7.17 (t, *J* = 7.7 Hz, 1H), 7.10 (dt, *J* = 8.4, 1.0 Hz, 1H), 6.95 (d, *J* = 8.1 Hz, 1H), 6.89 (d, *J* = 8.2 Hz, 1H), 6.78 (td, *J* = 7.4, 0.8 Hz, 1H), 6.63 (dd, *J* = 7.5, 1.7 Hz, 1H), 6.51 (d, *J* = 7.7 Hz, 1H), 5.70 (p, *J* = 6.7 Hz, 1H), 3.16 (dq, *J* = 14.9, 7.4 Hz, 1H), 2.69 (dq, *J* = 15.0, 7.4 Hz, 1H), 2.20 – 2.01 (m, 3H), 1.87 (dd, *J* = 7.6, 6.7 Hz, 6H), 1.79 (s, 3H), 1.60 (t, *J* = 7.4 Hz, 3H), 1.54 (s, 1H), 1.50 (s, 3H), 1.36 (s, 3H), 1.21 (s, 3H), 0.73 (t, *J* = 7.4 Hz, 3H).

^{13}C NMR (101 MHz, CD_2Cl_2) δ 272.5, 249.2, 141.9, 141.7, 140.9, 139.2, 132.2, 130.3, 128.7, 128.5, 126.8, 126.0, 125.7, 124.0, 122.8, 122.2, 114.6, 82.4, 79.4, 68.3, 54.9, 54.5, 54.4, 54.3, 54.0, 53.7, 53.5, 52.4, 32.8, 32.5, 30.7, 28.8, 26.1, 26.0, 23.2, 22.8, 22.2, 15.4, 12.5.

IR $\tilde{\nu}$: 3064, 2973, 2934, 2876, 1807, 1738, 1712, 1646, 1588, 1576, 1524, 1452, 1421, 1387, 1370, 1331, 1298, 1276, 1237, 1194, 1152, 1129, 1111, 1090, 1061, 1034, 988, 921, 874, 841, 810, 780, 741, 707, 566, 522 cm^{-1}.

HRMS (ESI TOF *m/z*) calculated for $C_{34}H_{41}Cl_2NORuS_2$ $[M]^+$: 715.1050, Found: 715.1036.

EA: calculated for $C_{34}H_{41}Cl_2NORuS_2$: C: 57.05; H: 5.77; Found C: 56.92; H: 5.79.

Synthesis of ruthenium complex Ru11

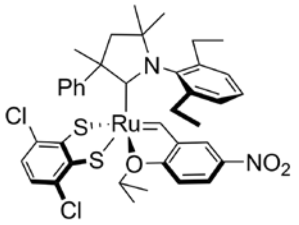

Following the general procedure, using **Ru7** (180 mg, 0.26 mmol, 1 equiv.), zinc dichlorodithiocatechol complex **1** (132 mg, 0.39 mmol, 1.5 equiv.), and THF (10 mL). The desired product was obtained as a brown solid (183 mg, 0.22 mmol, 85% yield). Contains some amount of *n*-hexane visible on ^{1}H and ^{13}C NMR.

^{1}H NMR (400 MHz, CD_2Cl_2) δ 13.85 (s, 1H), 8.17 (dd, *J* = 9.2, 2.7 Hz, 1H), 7.73 (d, *J* = 7.6 Hz, 2H), 7.59 – 7.45 (m, 3H), 7.42 (q, *J* = 2.4 Hz, 2H), 7.17 (t, *J* = 7.7 Hz, 1H), 7.07 (d, *J* = 9.0 Hz, 1H), 6.98 (d, *J* = 8.1 Hz, 1H), 6.90 (d, *J* = 8.2 Hz, 1H), 6.46 (d, *J* = 7.6 Hz, 1H), 4.20 (s, 1H), 3.18 (dq, *J* = 14.9, 7.4 Hz, 1H), 2.94 (d, *J* = 13.1 Hz, 1H), 2.71 (dq, *J* = 15.2, 7.4 Hz, 1H), 2.34 (d, *J* = 13.1 Hz, 1H), 2.10 (s, 3H), 1.64 (t, *J* = 7.3 Hz, 3H), 1.56 (s, 3H), 1.43 (d, *J* = 6.5 Hz, 3H), 1.29 (s, 6H), 0.89 (t, *J* = 6.8 Hz, 2H), 0.87 (q, *J* = 7.1 Hz, 3H).

^{13}C NMR (101 MHz, CD_2Cl_2) δ 269.1, 248.5, 158.4, 152.8, 144.7, 142.2, 141.4, 140.9, 140.7, 139.2, 139.0, 131.2, 129.8, 129.5, 128.6, 127.7, 127.6, 126.8, 125.3, 123.6, 122.6, 121.9, 119.0, 113.7, 83.7, 79.2, 62.3, 49.8, 31.6, 30.4, 29.7, 29.7, 25.0, 23.4, 22.6, 22.0, 20.8, 14.6, 13.9, 12.4.

IR $\tilde{\nu}$: 3076, 2974, 2931, 2877, 1599, 1574, 1519, 1469, 1444, 1415, 1392, 1374, 1339, 1265, 1227, 1201, 1159, 1134, 1105, 1085, 1062, 1032, 987, 948, 912, 895, 830, 813, 755, 745, 696, 664, 653, 600, 529 cm^{-1}.

HRMS (ESI TOF *m/z*) calculated for $C_{39}H_{41}Cl_2N_2O_3RuS_2$ $[M\text{-}H]^+$: 821.0979, Found: 821.0957.

EA: calculated for $C_{39}H_{42}Cl_2N_2O_3RuS_2 + CH_2Cl_2$: C: 52.92; H: 4.89; Found C: 52.63; H: 4.72.

Synthesis of ruthenium complex Ru12

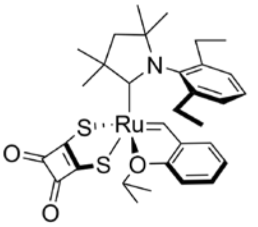

Following the general procedure, using **Ru6** (200 mg, 0.35 mmol, 1 equiv.), squaric acid derivative **2** (140 mg, 0.52 mmol, 1.5 equiv.), and THF (15 mL). The desired product was obtained as a brown solid (205 mg, 0.32 mmol, 91% yield).

^{1}H NMR (400 MHz, CD_2Cl_2) δ 14.85 (d, *J* = 0.9 Hz, 1H), 7.49 – 7.36 (m, 2H), 7.22 (t, *J* = 7.7 Hz, 1H), 7.14 (d, *J* = 8.5 Hz, 1H), 6.87 (t, *J* = 7.4 Hz, 1H), 6.73 (dd, *J*

= 7.6, 1.7 Hz, 1H), 6.51 (d, *J* = 7.7 Hz, 1H), 5.62 (hept, *J* = 6.8 Hz, 1H), 2.91 (dq, *J* = 15.0, 7.5 Hz, 1H), 2.58 (dq, *J* = 14.9, 7.4 Hz, 1H), 2.13 (s, 2H), 2.01 (dq, *J* = 14.8, 7.3 Hz, 1H), 1.93 (s, 3H), 1.80 (t, *J* = 6.9 Hz, 6H), 1.66 (s, 3H), 1.49 (t, *J* = 7.4 Hz, 3H), 1.32 (s, 3H), 1.19 (s, 3H), 0.74 (t, *J* = 7.4 Hz, 3H).

^{13}C NMR (101 MHz, CD_2Cl_2) δ 270.1, 262.1, 223.9, 207.1, 189.0, 187.5, 154.9, 141.2, 141.1, 140.2, 137.9, 130.3, 129.4, 127.2, 126.4, 126.0, 123.4, 115.4, 84.9, 79.8, 55.7, 52.4, 32.8, 32.5, 30.8, 28.5, 26.0, 23.3, 22.4, 21.7, 15.0, 12.5.

IR $\tilde{\nu}$: 3063, 2970, 2933, 2875, 1806, 1738, 1711, 1646, 1589, 1576, 1521, 1464, 1453, 1429, 1421, 1388, 1328, 1298, 1238, 1194, 1150, 1129, 1111, 1089, 1033, 987, 921, 904, 874, 811, 781, 740, 708, 567, 521 cm^{-1}.

HRMS (ESI TOF *m/z*) calculated for $C_{32}H_{40}NO_3RuS_2$ $[M+H]^+$: 652.1493, Found: 652.1488.

EA: calculated for $C_{32}H_{39}NO_3RuS_2$ + 0.25×CH_2Cl_2: C: 57.63; H: 5.92; Found C: 57.59; H: 6.17.

Cross-Metathesis Reactions

Cross-Metathesis reaction between 1-dodecene and (*Z*)-2-butene-1,4-diol—optimization

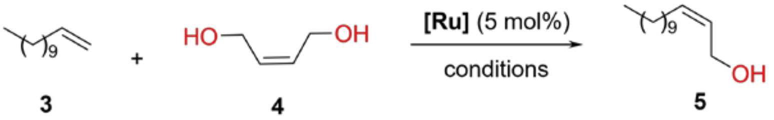

To the solution of 1-dodecene (**3**) (583 mg, 0.77 mL, 3.5 mmol) and (*Z*)-2-butene-1,4-diol (**4**) (642 mg, 0.6 mL, 7.0 mmol) in a dry solvent (THF or DCM, 34 mL) 5 mol% of a corresponding catalyst (**Ru8**, **Ru10** or **Ru4b**) was added in one portion in argon flow. The resulting mixture was stirred under given conditions (see Table 1) for 4 h. After the reaction time was completed, the mixture was quenched with SnatchCat (4.4 equiv. *vs* Ru). All volatiles were removed under reduced pressure and the crude product was purified using column chromatography (SiO_2, eluent: from *n*-hexane to 10% EtOAc/*n*-hexane). The product was obtained as a colorless oil.

Table S1. Conditions of CM reaction of 1-dodecene (**3**) and (*Z*)-2-butene-1,4-diol (**4**), conversion of 1-dodecene (**3**), yield, and *Z*/*E* ratio of the CM product.

Catalyst	Conditions	Conversion of **3**, %	Yield of **5**, %	*Z*/*E* ratio
**Ru4a**	THF, RT	64	56	95:5
**Ru4b**	THF, RT	64	50	95:5
**Ru10**	THF, RT	37	30	95:5
**Ru10**	THF, 50 °C	35	22	95:5
**Ru10**	THF, 80 °C	42	17	95:5
**Ru10**	DMC, RT	55	53	97:3
**Ru10**	DMC, 80 °C	44	not isolated	nd
**Ru8**	THF, RT	7	0	nd
**Ru8**	THF, 50 °C	11	0	nd
**Ru8**	THF, 80 °C	11	0	nd

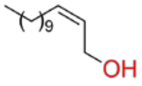

^{1}H NMR (400 MHz, $CDCl_3$) δ (ppm) for *Z* isomer (major): 5.76 – 5.48 (m, 2H), 4.09 (dd, *J* = 5.6, 0.9 Hz, 2H), 2.08 – 2.01 (m, 2H), 1.26 (m, 16H), 0.89 – 0.86 (m, 3H).

^{13}C NMR (101 MHz, $CDCl_3$) δ (ppm) 133.7, 128.8, 63.9, 32.2, 31.9, 29.6, 29.5, 29.3, 29.2, 29.2, 29.1, 22.7, 14.1.

^{1}H and ^{13}C NMR spectra are in agreement with those previously reported.[1]

Cross-Metathesis reaction of 1-dodecene and (*Z*)-2-butene-1,4-diol in DMC

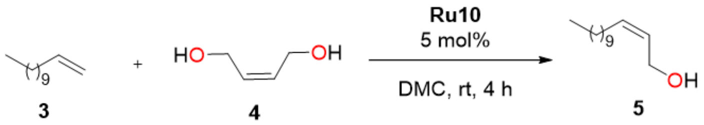

Preparation of stock solutions in DMC

In a 5 mL volumetric flask 1.25 mmol of 1-dodecene (210.4 mg, 278 µL), 2.5 mmol of butene-1,4-diol (220.3 mmol, 206 µL) and 0.6 mmol of tetradecane (used as an internal standard, 163 µL) were mixed.

Then, the flask was filled up to the volume mark (approx. 4.4 mL of DMC). An aliquot (0.2 mL) of this solution was used for GC.

Procedure of the CM reaction of 1-dodecene and butene-1,4-diol in DMC

An aliquot (0.8 mL) of a stock solution was placed in 4 mL vials (4 vials, 0.8 mL in each). Then, 7.2 mg of **Ru10** was added in one portion to each of the vials. The reaction mixture in the first vial was stirred without pyridine. To the 2nd vial 0.8 µL of pyridine was added using Hamilton syringe. To the 3rd vial - 1.6 µL of pyridine and to the 4th vial - 4 µL of pyridine was added. The reaction mixtures were stirred for 4 h at room temperature in the glovebox. After the reaction time was completed, aliquots from each vial (0.2 mL) were taken, the conversion of 1-dodecene was determined by GC. For the **entry 1** the mixture was quenched with **SnatchCat** (4.4 equiv. *vs* Ru). All volatiles were removed under reduced pressure and the crude product was purified using column chromatography (SiO_2, eluent: from *n*-hexane to 10% EtOAc/*n*-hexane). The product was obtained as a colorless oil (21 mg, 53%).
Note: **Ru10** was not soluble in a given solvent, therefore it was used added as a solid.

Table S2. Conversion of 1-dodecene in DMC

Entry	Pyridine (equiv. *vs* [Ru])	Conversion of **3**, %	Yield of **5**, %	*Z*/*E* ratio
1	-	54*	53	97:3
2	1	13	not isolated	nd
3	2	26	not isolated	nd
4	5	18	not isolated	nd

Cross metathesis reaction of 1-dodecene and (*Z*)-2-butene-1,4-diol in THF

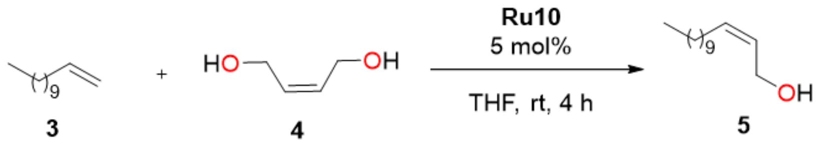

Preparation of stock solutions in THF

A. In a 5 mL volumetric flask 1.25 mmol of 1-dodecene (210.4 mg, 278 µL), 2.5 mmol of butene-1,4-diol (220.3 mmol, 206 µL) and 0.6 mmol of tetradecane (used as an internal standard, 163 µL) were mixed. Then, the flask was filled up to the volume mark (approx. 4.4 mL of THF). An aliquot (0.2 mL) of this solution was used for GC.

B. Ru complex **Ru10** (35.8 mg) was dissolved in 1 mL of THF in a 4 mL vial.

Procedure of the CM reaction of 1-dodecene and butene-1,4-diol in THF

An aliquot (0.8 mL) of a stock solution **A** was placed in 4 mL vials (4 vials, 0.8 mL in each). Then, 0.2 mL of stock solution of **Ru10** complex (0.05 mmol/0.2 mL) was added to each of the vials.

The reaction mixture in the first vial was stirred without pyridine. To the 2nd vial 0.8 μL of pyridine was added using Hamilton syringe. To the 3rd vial - 1.6 μL of pyridine and to the 4th vial - 4 μL of pyridine was added. The reaction mixtures were stirred for 4 h at room temperature in the glovebox. After the reaction time was completed, aliquots from each vial (0.2 mL) were taken, the conversion of 1-dodecene was determined by GC.

Table S3. Conversion of 1-dodecene in THF

Entry	Pyridine (equiv. *vs* [Ru])	Conversion of **3**, %
1	-	36
2	1	27
3	2	40
4	5	34

Cross-Metathesis reaction between 1-dodecene and (*Z*)-1,4-diacetoxy-2-butene or (*Z*)-3-hexene

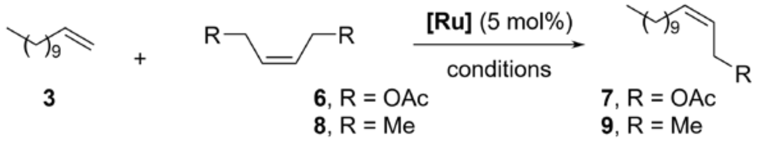

To the solution of 1-dodecene (**3**) (33.7 mg, 44 μL, 0.2 mmol) and a corresponding internal olefin (*cis*-1,4-diacetoxy-2-butene (68.8 mg, 64 μL, 0.4 mmol) or *cis*-3-hexene (33.7 mg, 0.4 mmol)) and tetradecane (0.5 equiv., used as an internal standard) in dry THF (1 mL) 5 mol% of a corresponding catalyst (**Ru8**, **Ru10** or **Ru4b**) was added in one portion in the glovebox. The resulting mixture was stirred at room temperature for 4 h. After the reaction time was completed, the mixture was quenched with SnatchCat (4.4 equiv. *vs* Ru). The conversion of **3** was determined by GC.

Self-Metathesis—Dimerization of 1-dodecene

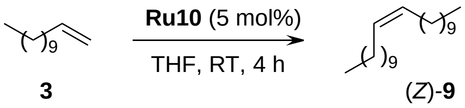

In the glovebox, to the 4 mL vials was placed solution of 1-dodecene (60 mg, 0.385 mmol, 1.0 equiv.), and tetradecane (36 mg, 0.179 mmol, 0.5 equiv.) in THF. Then solution of catalyst **Ru4a** or **Ru10** was added to obtain concentration of 1-dodecene (0.2M) and cat loading 5 mol%. Reactions was stirred in RT for 4 h. Then crude reaction mixture was analyzed by GC. No product was observed.

Self-Metathesis—Dimerization of hex-3-ene-1-ol

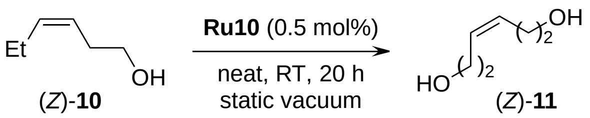

Cis-3-hexene-1-ol (280 mg, 0.33 mL, 2.8 mmol) and ruthenium complex **Ru10** (0.5 mol%) were placed in a Schlenk flask and stirred neat at room temperature at 800 mbar for 20 h (to remove the emerging hex-3-ene byproduct). Reaction control *via* TLC or GC did not indicate any conversion of substrate.

Self-Metathesis—Disproportionation of oleic acid methyl ester

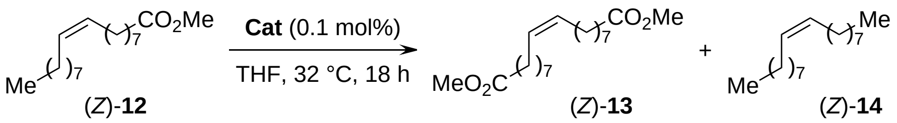

In the glovebox, to the 4 mL vials equipped with a magnetic stirring bar, a solution of methyl oleate (135 mg, 0.42 mmol, 1.0 equiv., 98% of Z isomer) in THF, tetradecane (0.1 equiv.) and a solution of a corresponding catalyst (0.1 mol%) were placed, such that the concentration of methyl oleate in the mixture was 0.1M. Reactions were carried out at room temperature for 18 hours. The conversion and the composition of the crude mixtures were determined by GC. In case of **Ru10** reaction reached 14% of conversion (both products give Z/E ratio 95:5). In case of **Ru4b** reaction has reached an equilibrium state (50% of conversion, both products give *Z*/*E* ratio 95:5).

Self-Metathesis—Disproportionation of elaidic acid methyl ester

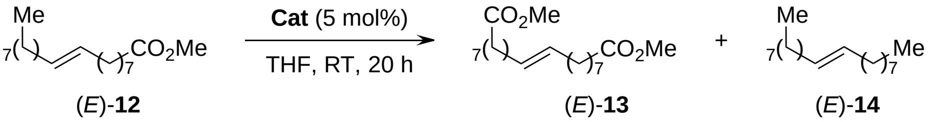

In the glovebox, to the 4 mL vials was placed solution of Methyl Elaidate (135 mg, 0.42 mmol, 1.0 equiv., 92% of E isomer), and tetradecane (17 mg, 0.084 mmol, 0.2 equiv.) in THF. Then solution of catalyst was added to obtain concentration of Methyl Elaidate (0.42M) and cat loading 5 mol%. Reactions was stirred in RT for 20h. Then crude reaction mixture was analyzed by GC. No product was observed in case of Ru10. In case of Ru4a reaction has reached an equilibrium state (50% of conversion, both products give *E*/*Z* ratio 95:5).

Ring-Closing Metathesis

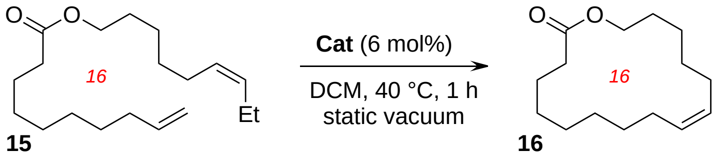

In the glovebox, a stock solution of (*Z*)-non-6-en-1-yl dec-9-enoate (1 mmol, 294 mg, 1 equiv.), Tetradecane (as internal standard; 0.1 mmol, 20 mg, 0.1 equiv.) in DCM (320 ml) was prepared.

In the glovebox to a 50 ml Schlenk flask equipped with a stir bar a stock solution of (*Z*)-non-6-en-1-yl dec-9-enoate (0.1 mmol, 29.4 mg, 1 equiv.), tetradecane (0.01 mmol, 2 mg, 0.1 equiv.) in DCM (32 ml) was placed followed with addition of solution of appropriate catalyst (0.006 mmol, 0.06 equiv.) in DCM (1 ml, final conc.: 3 mM). The flask was sealed and taken out of the glovebox. After one freeze, pump, thaw cycle, the reaction flask was heated in oil bath at 40 °C for 1 h and then quenched with 1 mL of SnatchCat. Solvents were removed in vacuo, and the product was subjected to GC analysis using tetradecane as internal standard.

Table S4. Amount of catalyst used in macroRCM reaction of diene **15**.

Entry	Catalyst	Mass of catalyst used
1	**Ru4b**	5.15 mg
2	**Ru10**	4.34 mg
3	**Ru8**	4.76 mg

Ring-Closing Metathesis at high concentration

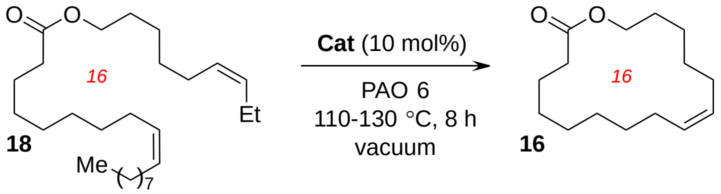

(*Z*)-6-nonenyl oleate (1.0 equiv.), 2.5 mL PAO6, and the appropriate amount of a corresponding catalyst (**Ru4b**, **Ru14**, **Ru10** or **Ru8**, 10 mol%) were charged to a reaction vessel equipped with a stirring bar. The vessel was fitted with a Hickmann cap, then connected to a diffusion pump (nominal pressure was 1x10^{-6} mbar) and placed into a heating bath. The reaction was carried out for another 8 hours at 110-130 °C. After completion of the reaction, the distillate was purified using column chromatography purification (SiO_2, using *n*-hexane followed by ethyl acetate as eluents). The fractions were combined and then concentrated on the rotary evaporator. As a result, a pure product was obtained in which the content of the *Z* isomer was determined by gas chromatography.

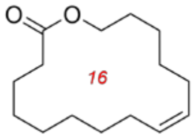

^{1}H NMR (400 MHz, $CDCl_3$) δ (ppm) *for a major isomer* 5.68 – 5.16 (m, 2H), 4.35 – 3.85 (m, 2H), 2.84 – 2.15 (m, 2H), 2.15 – 1.90 (m, 4H), 1.68 – 1.52 (m, 4H), 1.51 – 1.07 (m, 12H).

^{13}C NMR (101 MHz, $CDCl_3$) δ 173.9, 130.2, 129.9, 64.4, 35.3, 28.9, 28.5, 28.2, 28.2, 28.1, 27.7, 27.4, 26.3, 26.2, 24.9.

^{1}H and ^{13}C NMR spectra are in agreement with those previously reported.[2]

Activity/stability test

Ruthenium complexes **Ru8** and **Ru10** were chosen for activity/stability tests in comparison with commercially available **Ru4b**.

In the glovebox, to the NMR tube equipped with septum cap a solution of a corresponding Ru complex (0.014 mmol) and anthracene (1.3 mg, 0.007 mmol) in THF-d_8 (0.7 mL) were placed. Then ^{1}H NMR was measured. After that, through the septum a corresponding dry and degassed olefin was added as neat using Hamilton syringe. Subsequently, the NMR measurements were conducted at the given times (see Table S3).

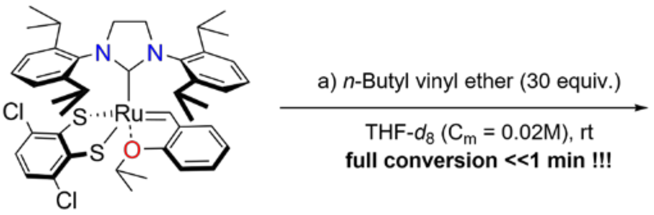

Ru4b

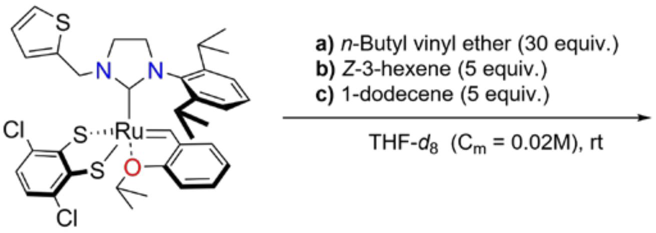

Ru8

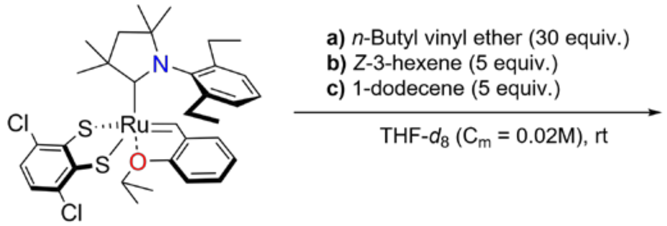

Ru10

a) The activation of the catalysts was tested in the reaction with *n*-butyl vinyl ether. Previously, Grubbs and co-workers assumed that the *stereoretentive* catalysts bearing symmetric NHC fully react with vinyl ether (<15 seconds in similar conditions in DCM).[2] In contest of our research we decided to use THF-d_8 instead. Symmetric NHC catalyst **Ru4b** (SIPr) gives full conversion within <<1 min, when **Ru8** in 1 min gives 79% of conversion. Interestingly, in 1 minute **Ru10** gives only 3%, and after 30 min only 47% of catalyst reacted with *n*-butyl-vinyl ether, to form a Fisher carbene [Ru]=CH(OBu) (**Ru13**). This experiment showed that new catalysts **Ru8** and **Ru10** are much more inert than those described already in literature, which partially explains unsuccessful results of the model reactions.

During second (**b**) and third (**c**) experiments we observed the behavior of catalysts in the presence of different types of olefins. Table below shows fading of the alkylidene signal during the time.

Table S5. Catalyst disappearance in the presence of various olefins over time.

Type of olefin	Time	% of catalyst disappearance	
		**Ru10**	**Ru8**
**Internal olefin** (*Z*)-3-hexene (**8**)	5 min	<1	<1
	1 h	4	18
	16 h	7	63
	24 h	9	73
	48 h	18	88
**Terminal Olefin** 1-dodecene (**3**)	1 min	5	8
	5 min	8	9
	15 min	12	17
	1 h	29	37
	3 h	46	64
	24 h	78	100

In both cases, catalysts were inert for both classes of olefins. We were not able to observe a new alkylidene signal of $14e^-$ species of ruthenium after activation step at any point of measurements. After comparison the results above with very poor yields of the products isolated from model reactions, we assume that the discussed catalysts activate extremely slowly, and, moreover, active spices have very short lifetime.

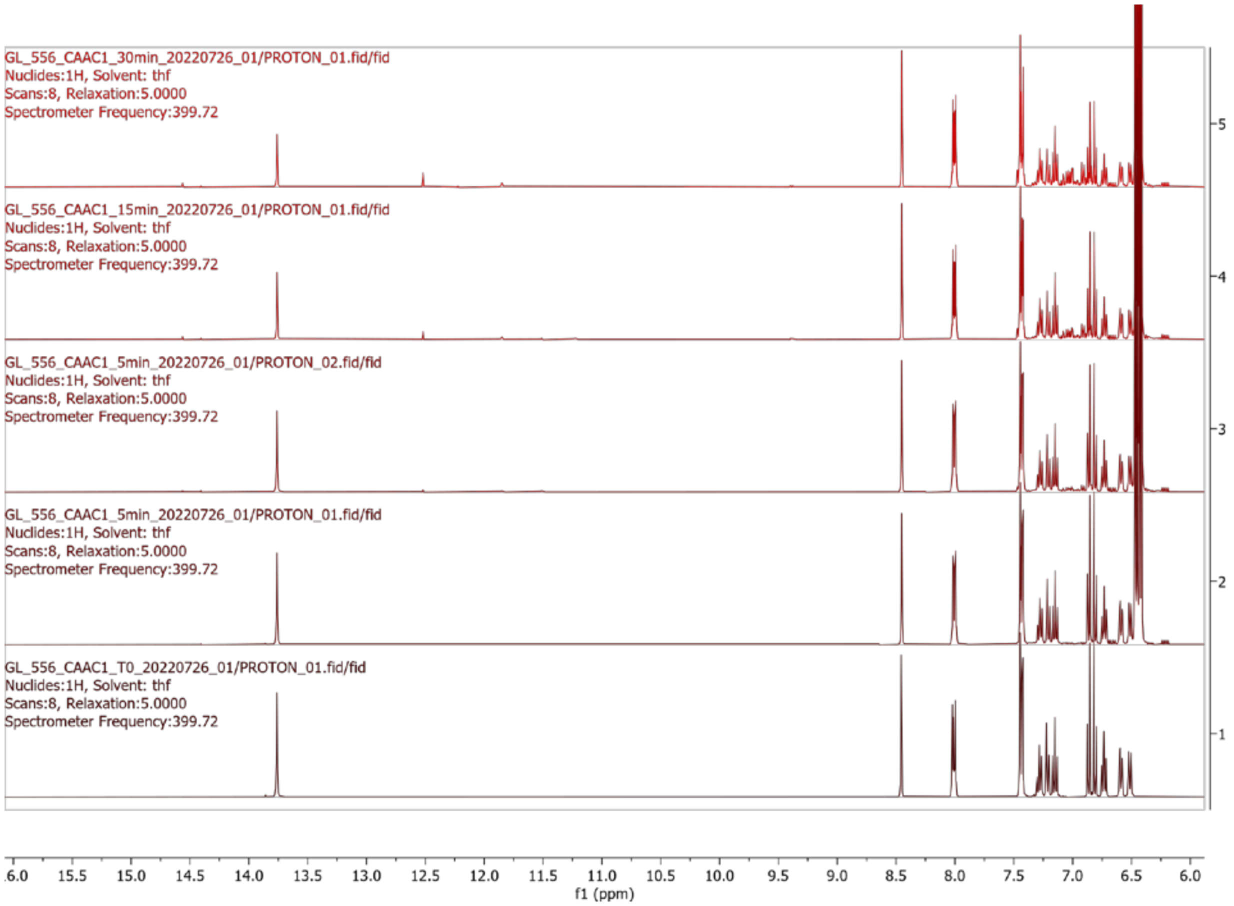

Figure S1. Stacked spectra from reaction with **Ru10** and *n*-butyl vinyl ether (**17**).

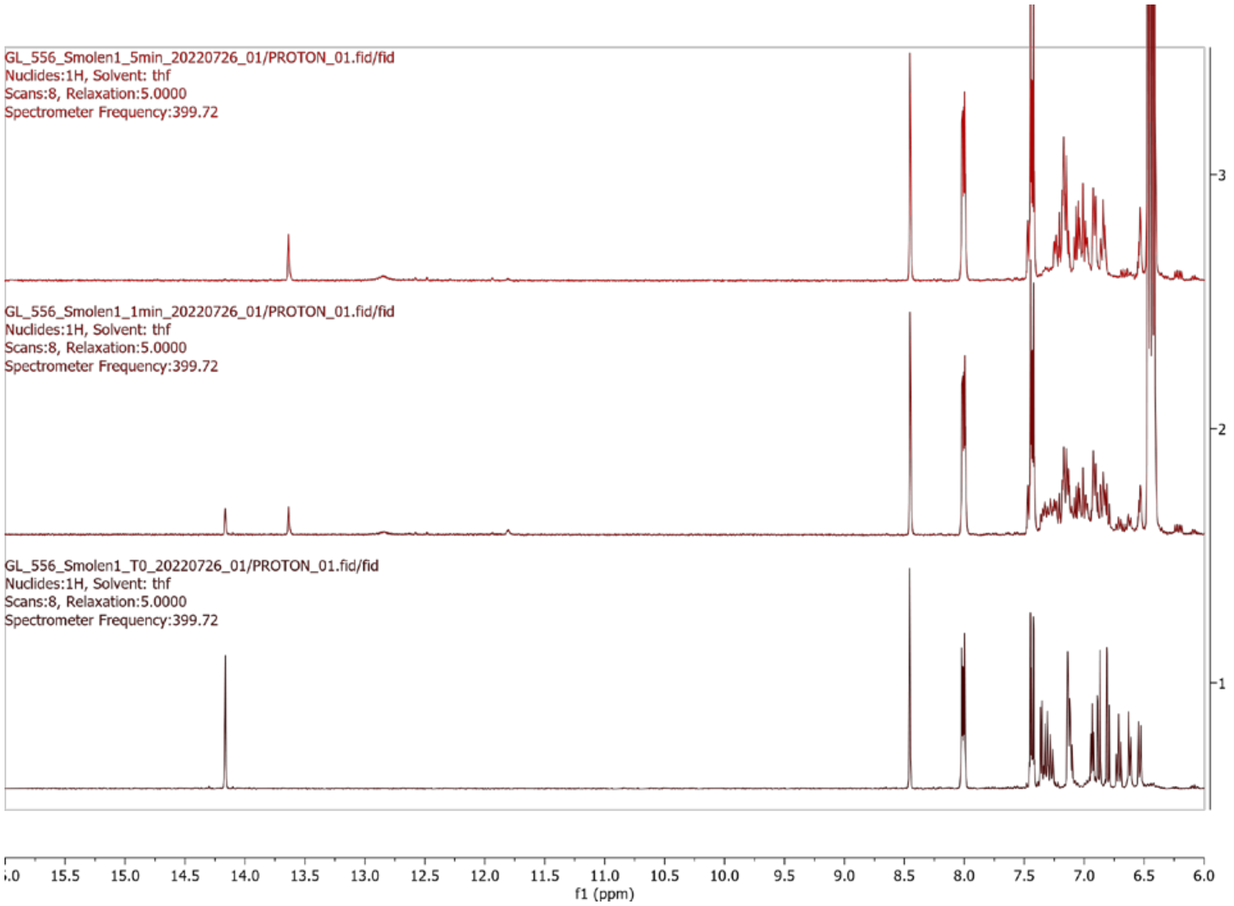

Figure S2. Stacked spectra from reaction with **Ru8** and *n*-butyl vinyl ether (**17**).

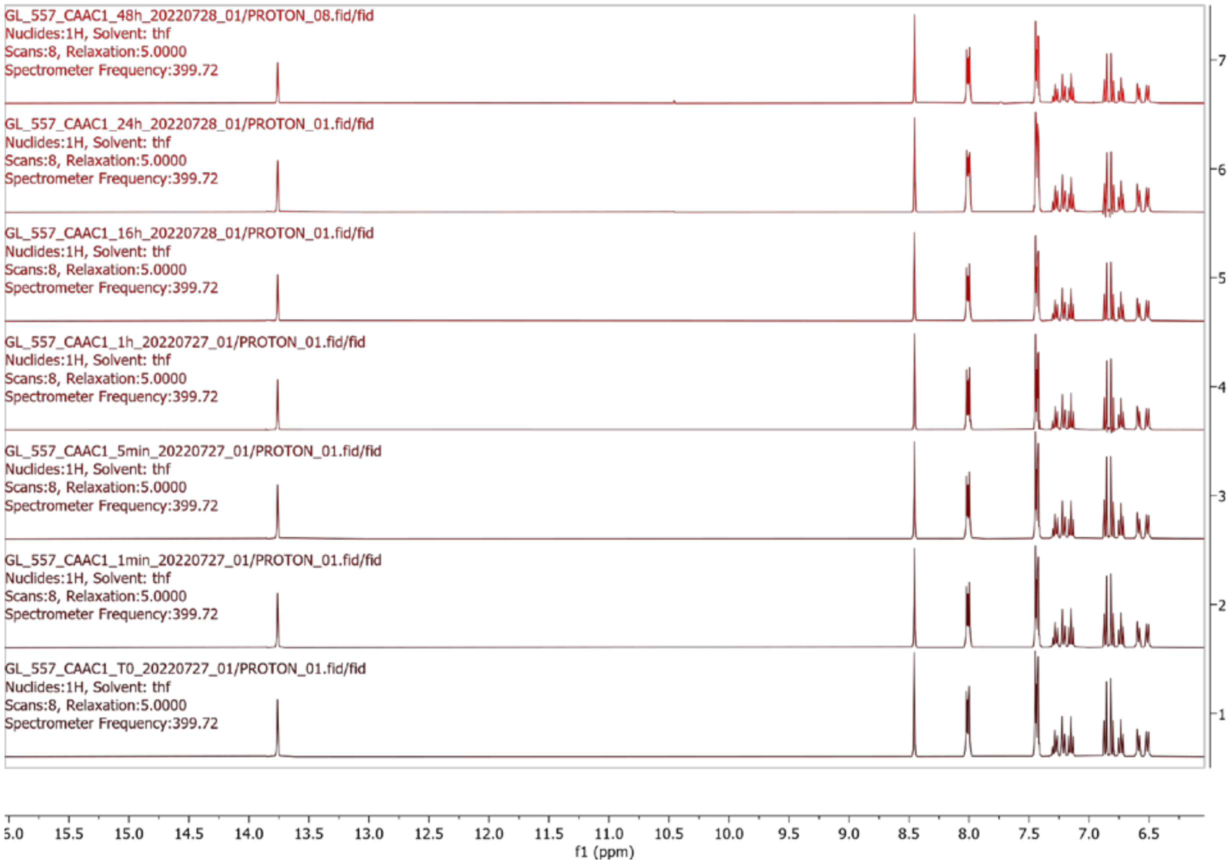

Figure S3. Stacked spectra from reaction with **Ru10** and (*Z*)-3-hexene (**8**).

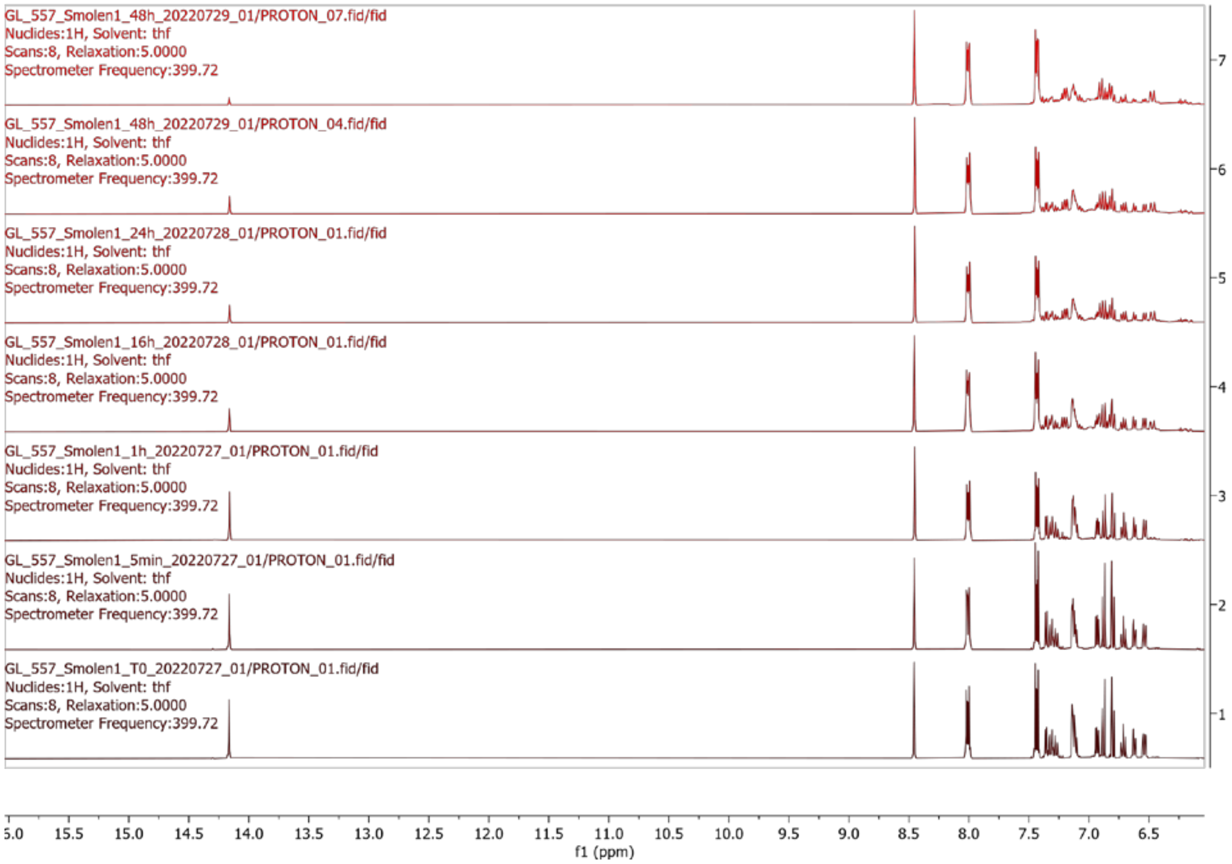

Figure S4. Stacked spectra from reaction with **Ru8** and (*Z*)-3-hexene (**8**).

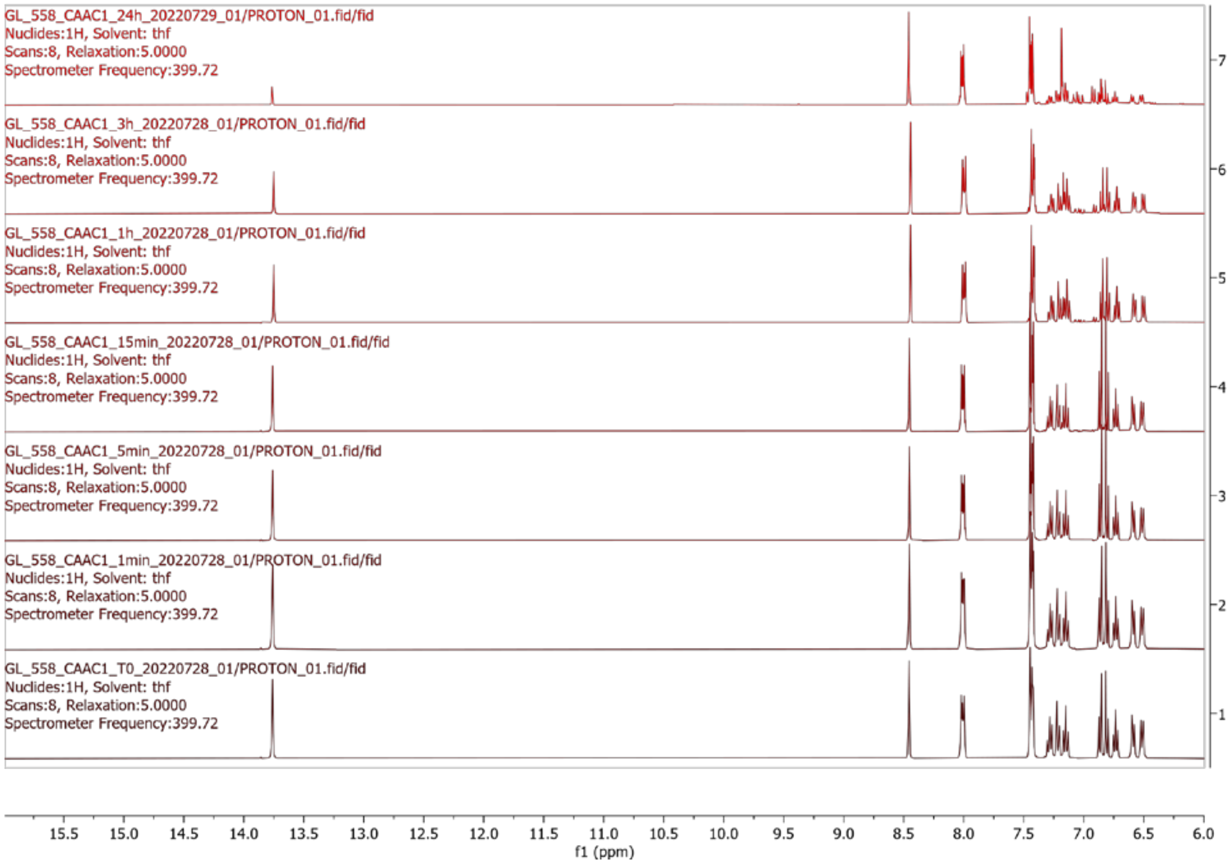

Figure S5. Stacked spectra from reaction with **Ru10** and 1-dodecene (**3**).

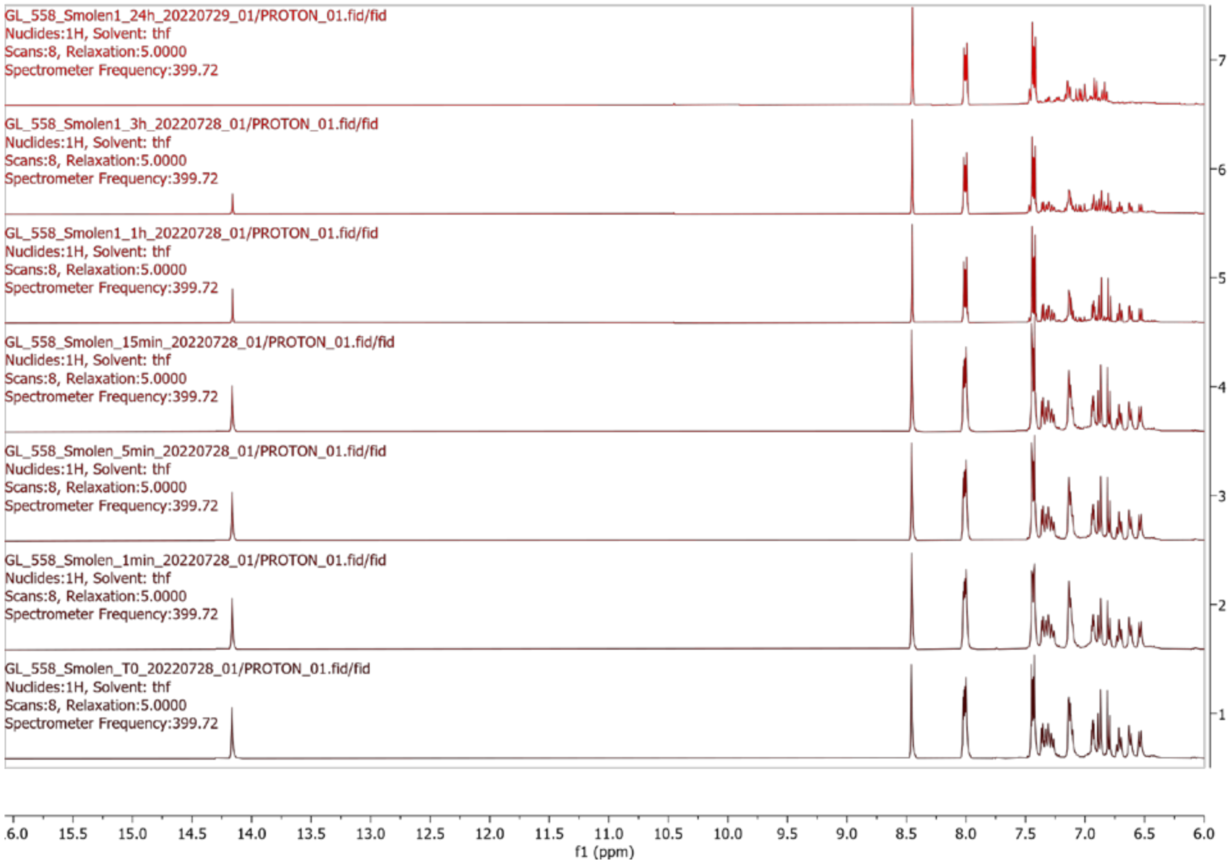

Figure S6. Stacked spectra from reaction with **Ru8** and 1-dodecene (**3**).

Copies of NMR Spectra

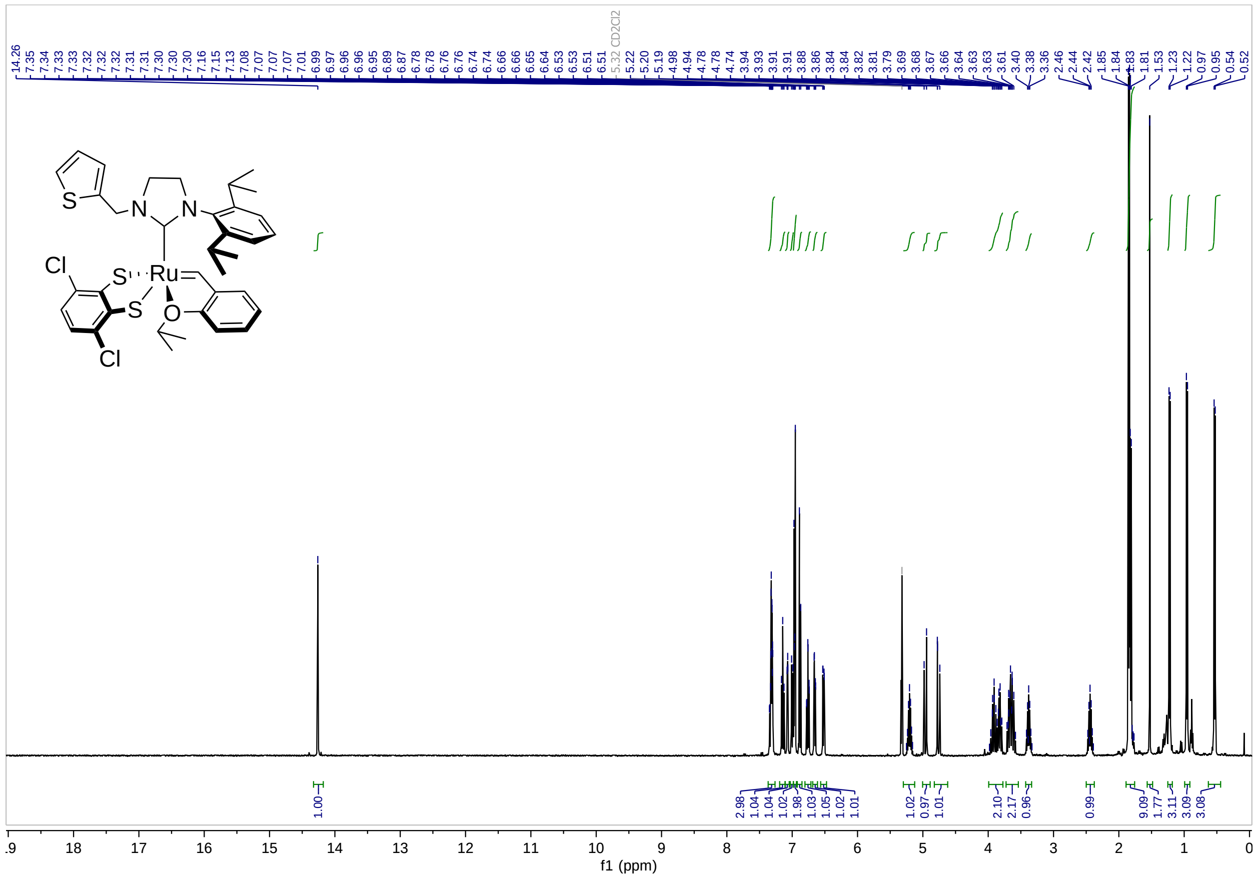

Figure S7. ^{1}H NMR spectrum of **Ru8**.

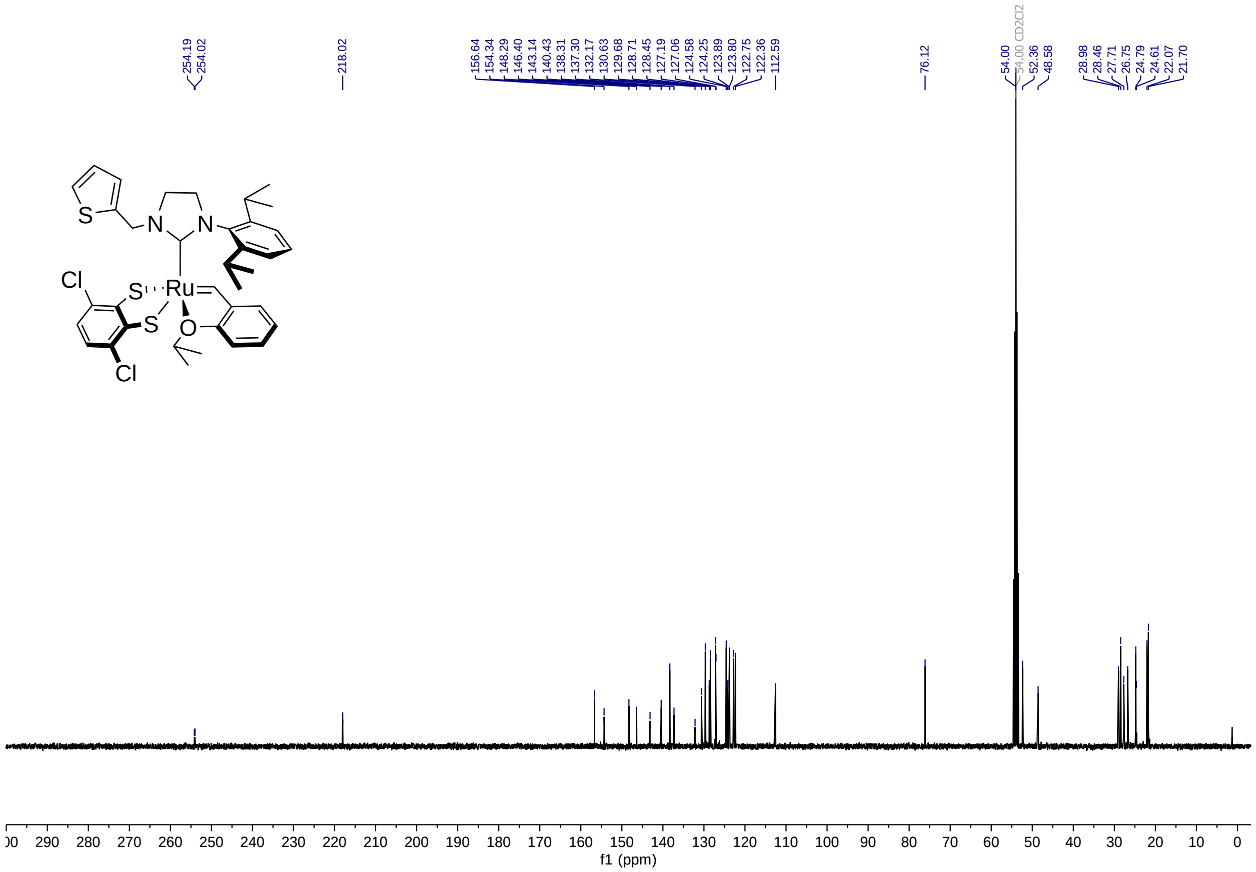

Figure S8. ^{13}C NMR spectrum of **Ru8**.

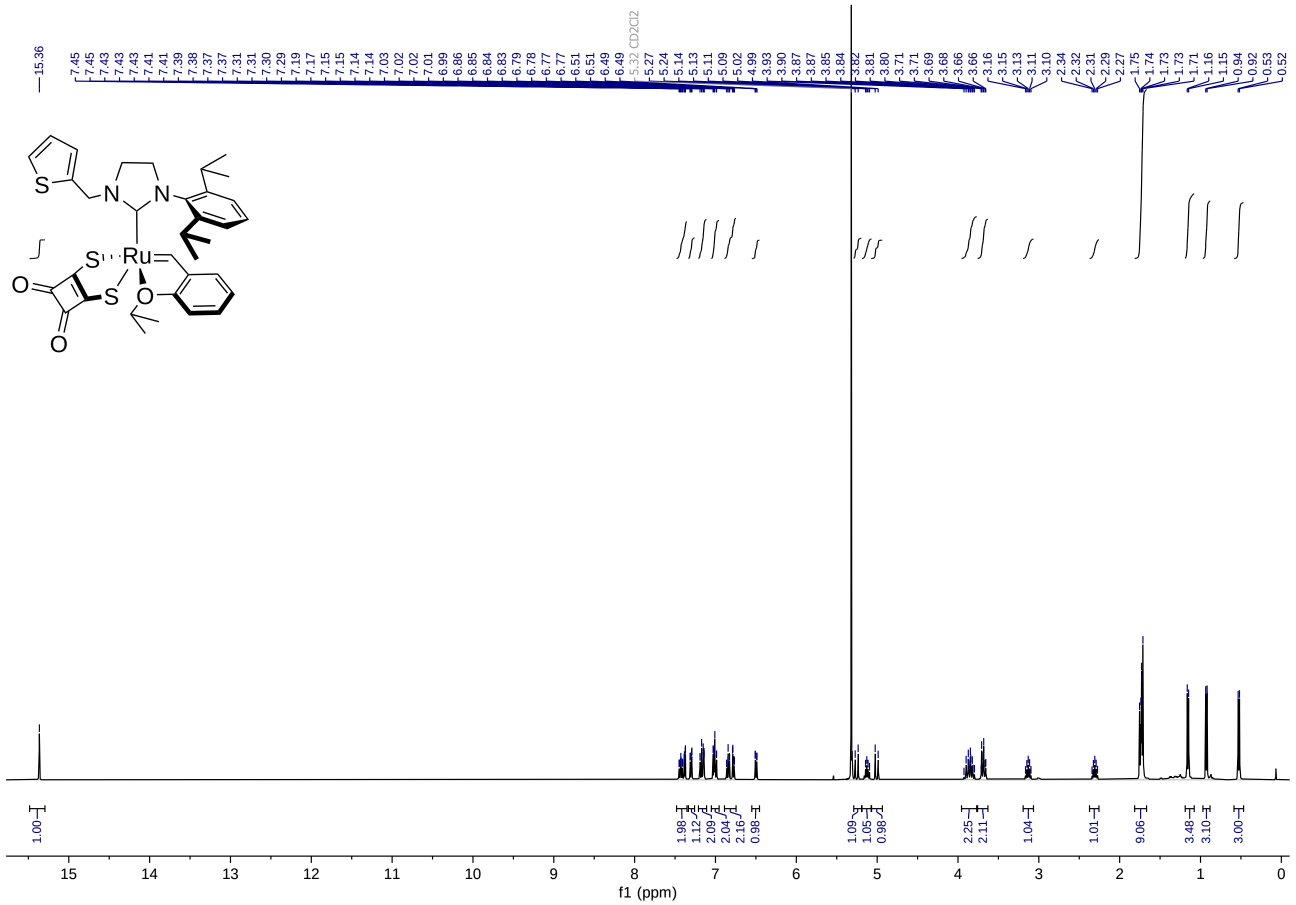

Figure S9. ^{1}H NMR spectrum of **Ru9**.

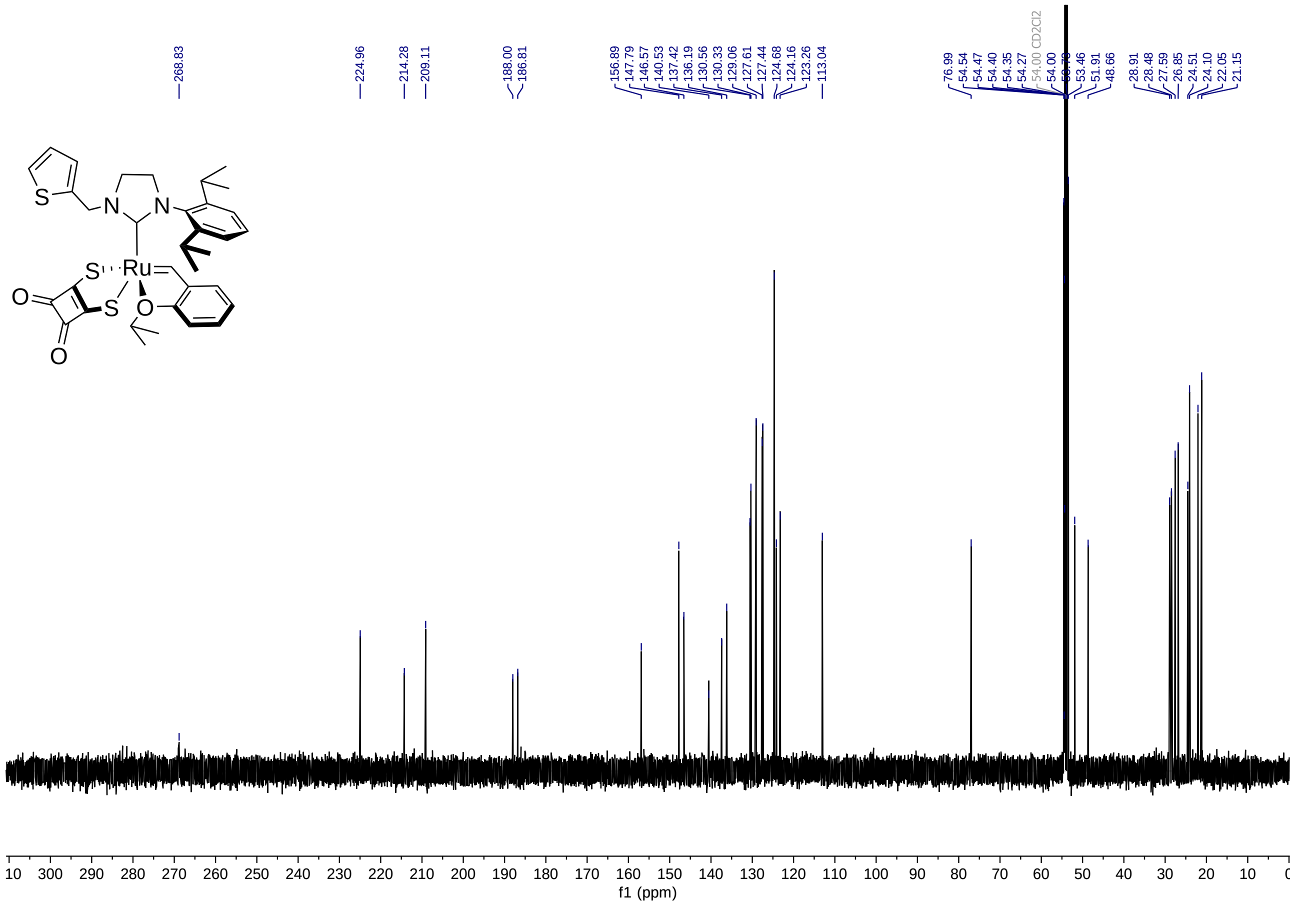

Figure S10. ^{13}C NMR spectrum of **Ru9**.

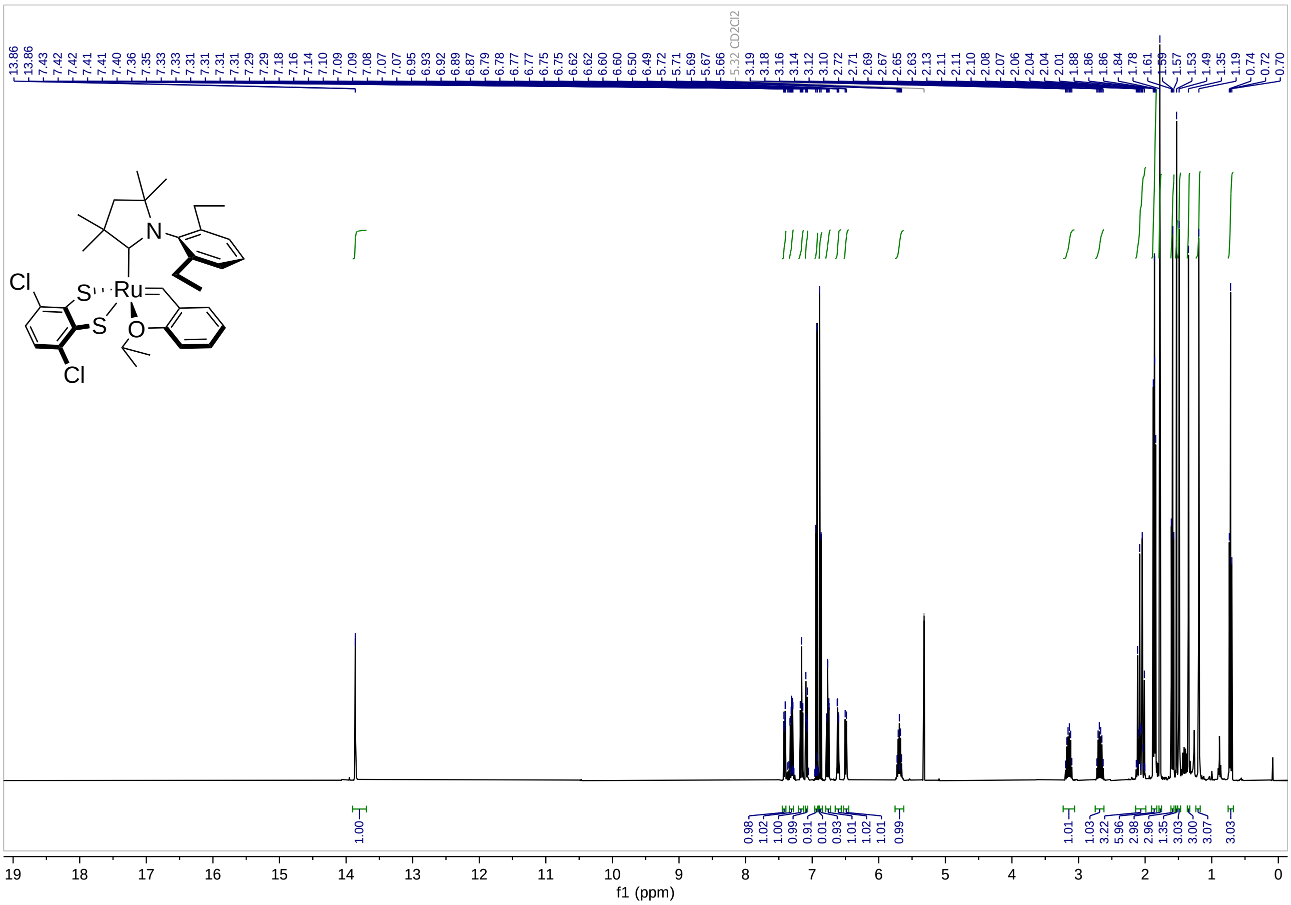

Figure S11. ^{1}H NMR spectrum of **Ru10**.

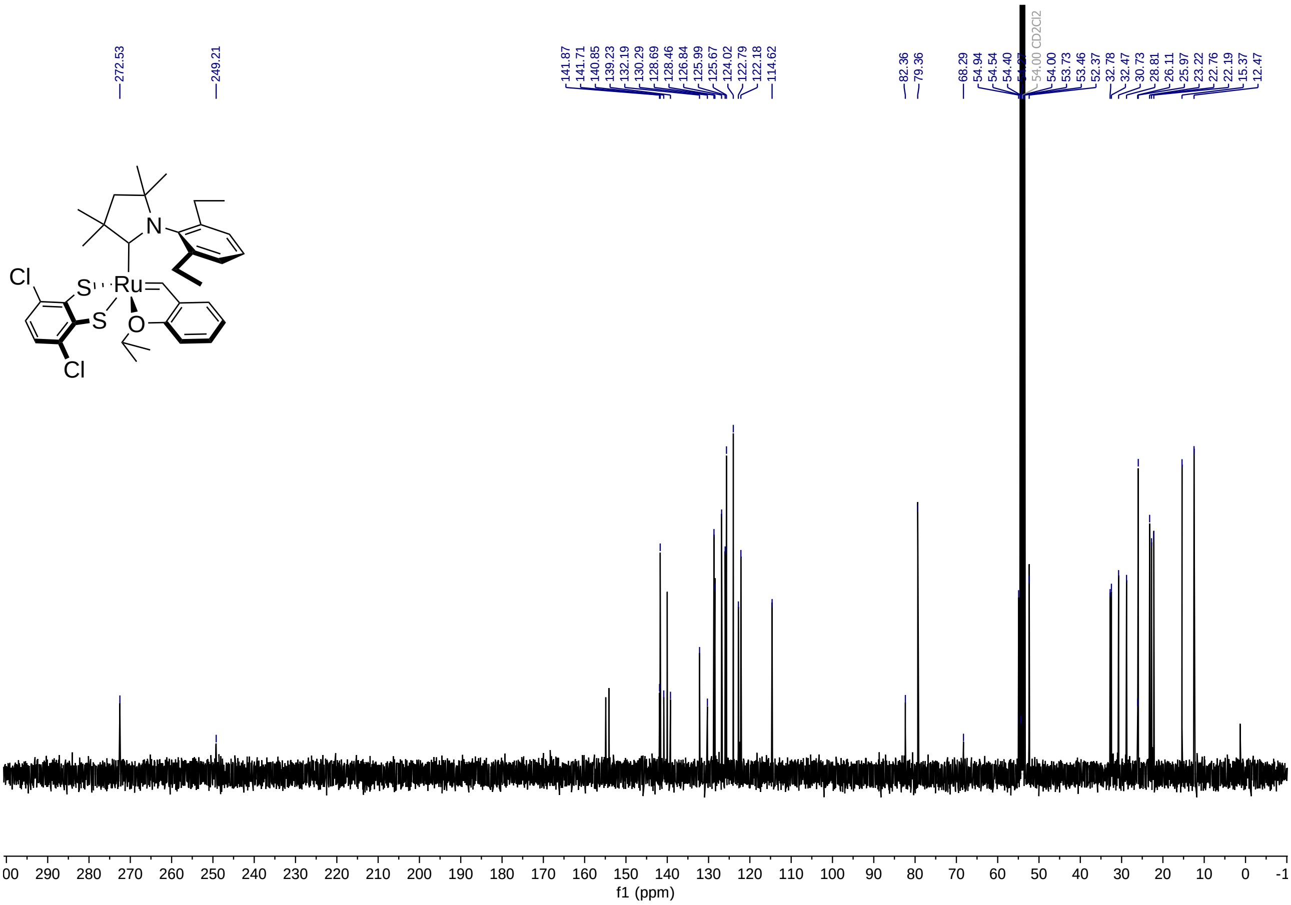

Figure S12. ^{13}C NMR spectrum of **Ru10**.

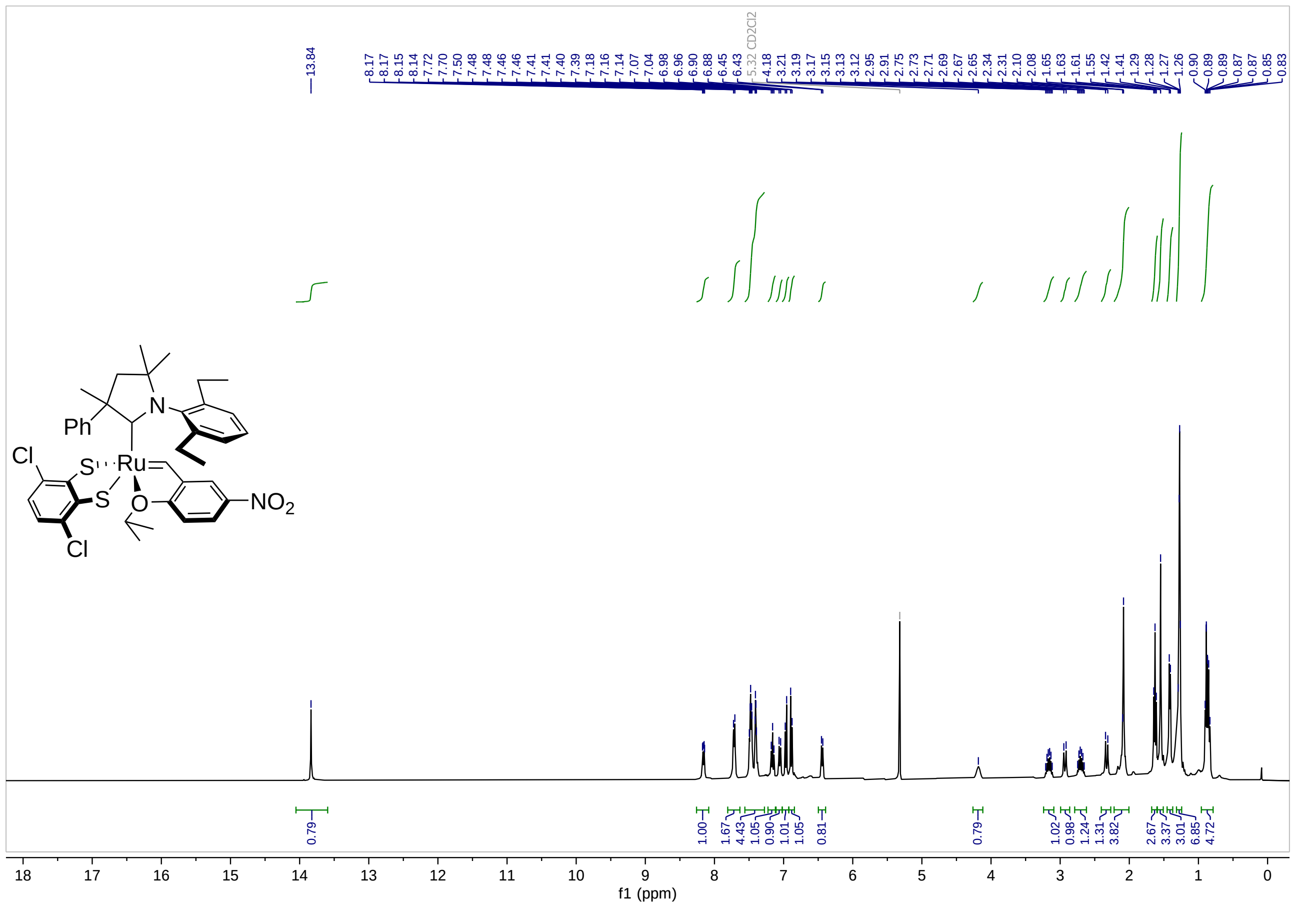

Figure S13. ^{1}H NMR spectrum of **Ru11**.

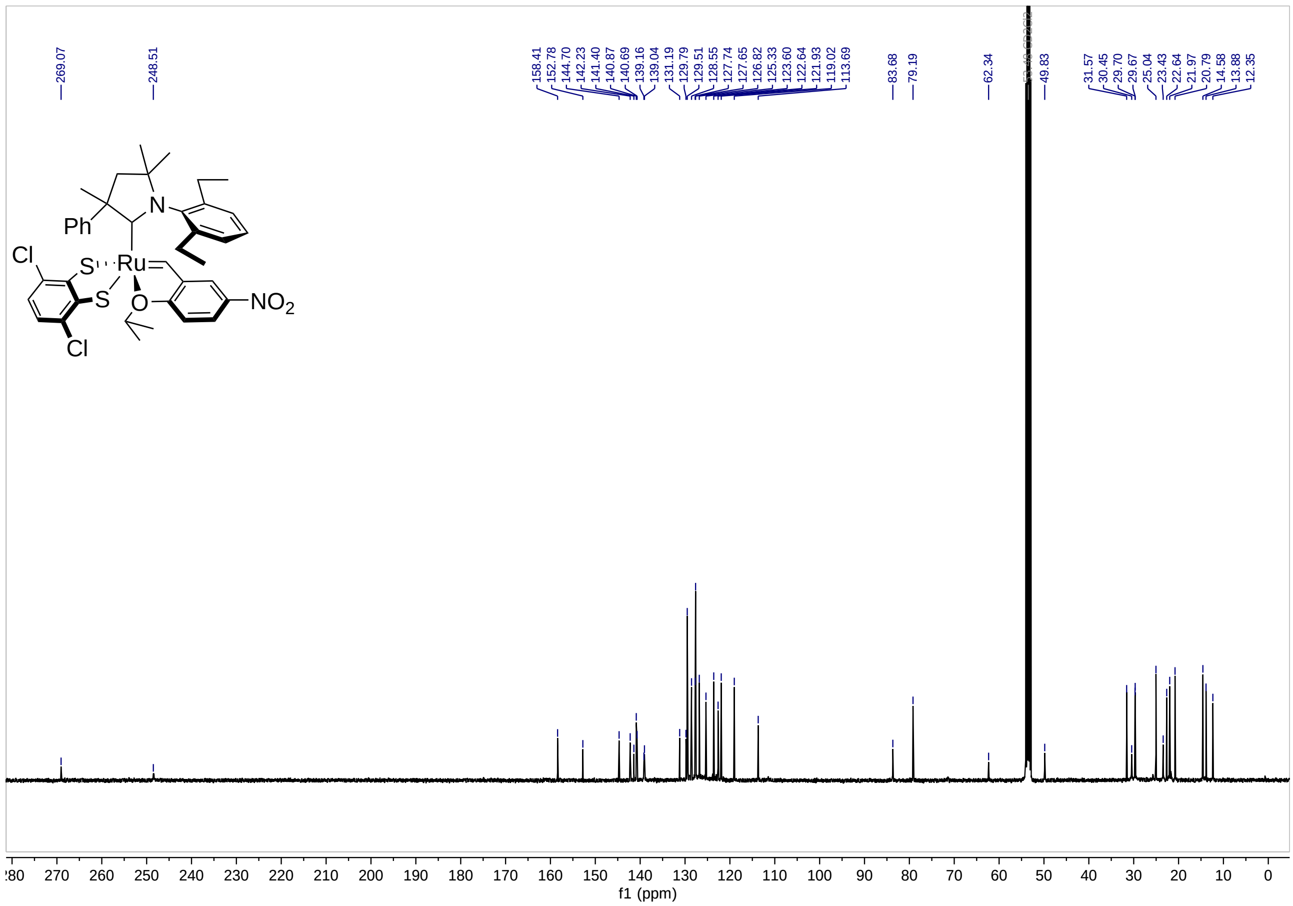

Figure S14. ^{13}C NMR spectrum of **Ru11**.

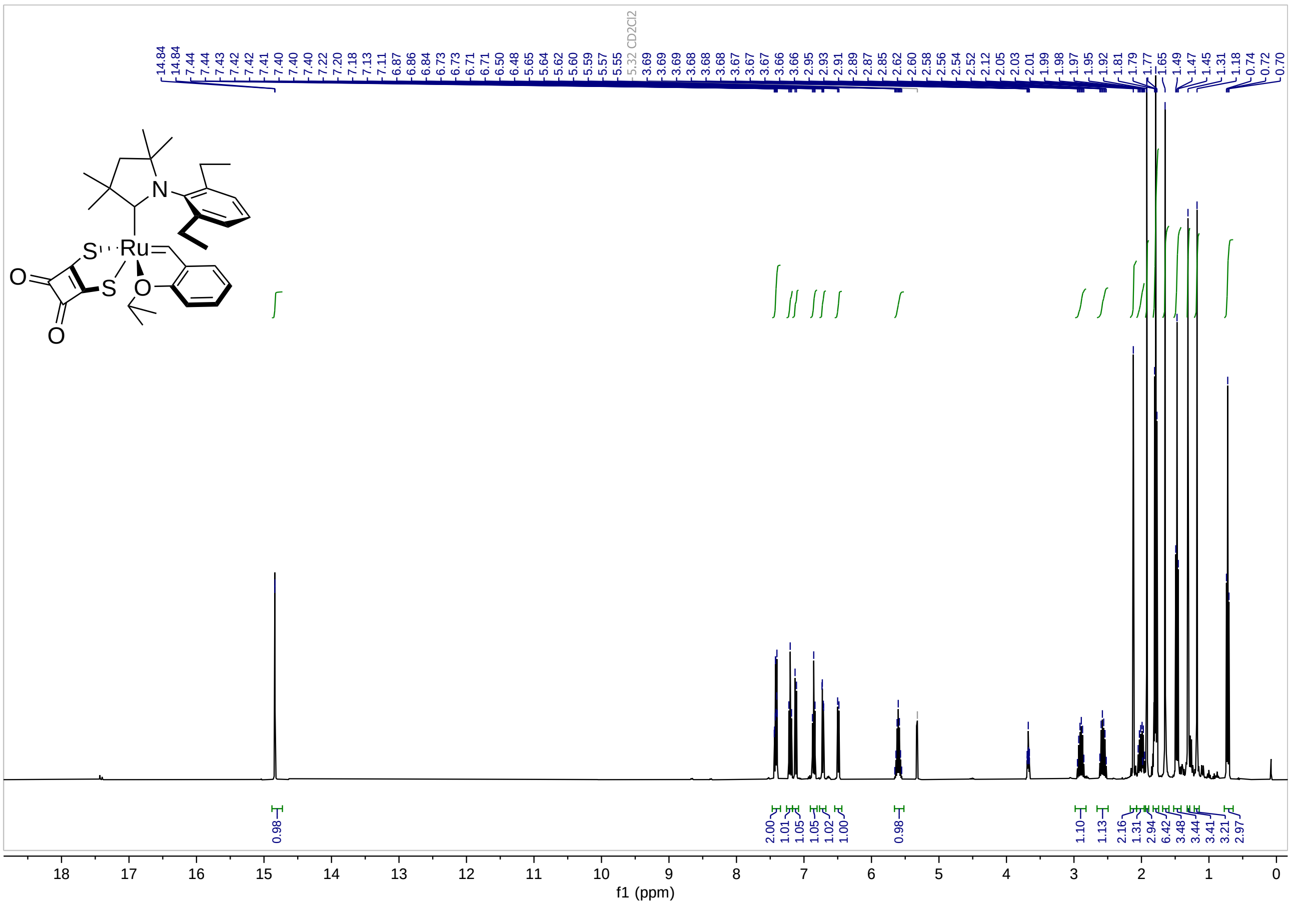

Figure S15. ^{1}H NMR spectrum of **Ru12**.

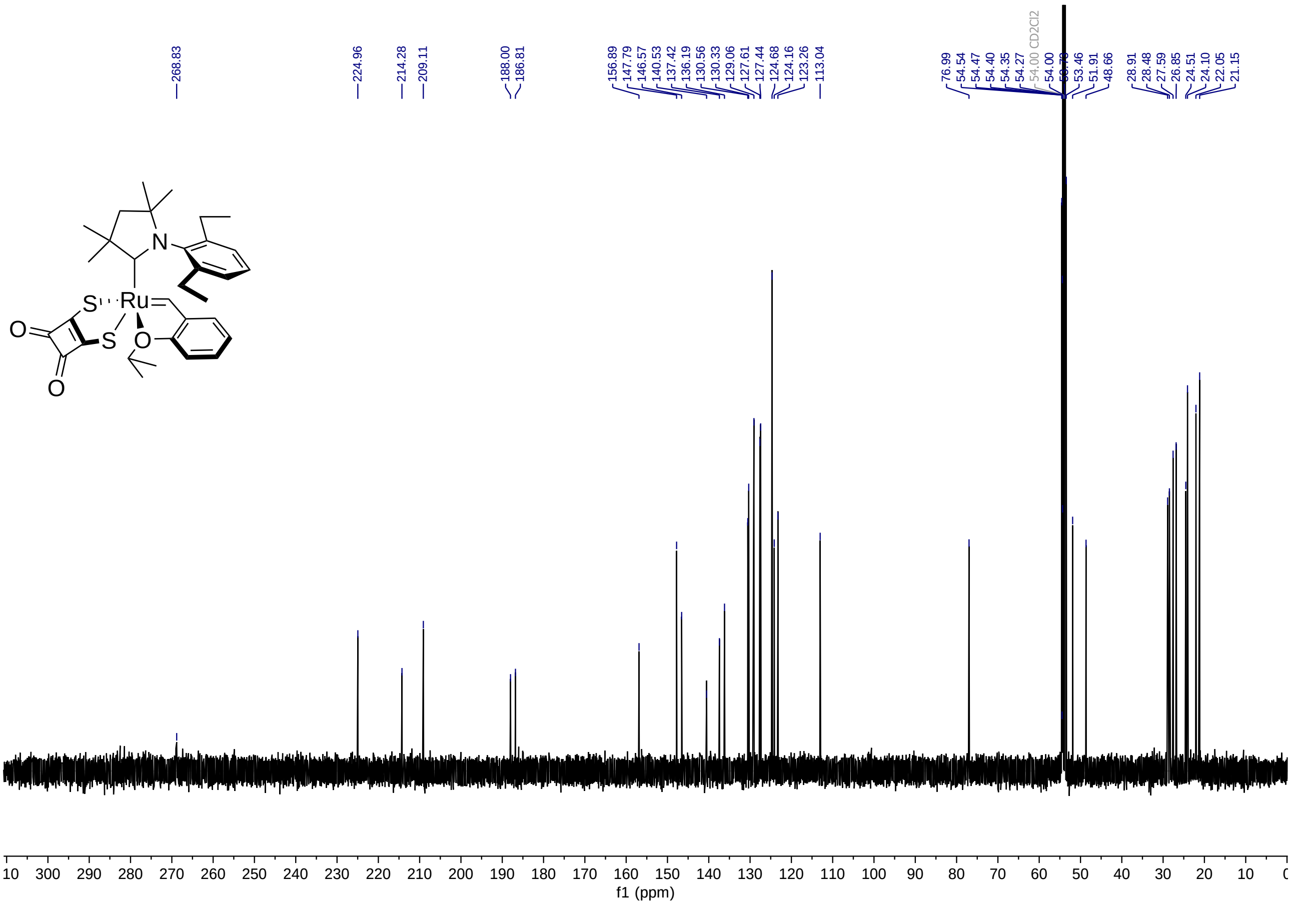

Figure S16. ^{13}C NMR spectrum of **Ru12**.

Crystallographic Information

Table S6. Summary of XRD analysis of **Ru8** and **Ru10**.

**Identification code**	**Ru8**	**Ru10**
Empirical formula	C37H43Cl4N2ORuS3	C34H41Cl2NORuS2
Formula weight	870.78	715.77
Temperature/K	100(2)	100(2)
Crystal system	monoclinic	monoclinic
Space group	P21/n	P21/n
a/Å	12.77849(18)	12.3241(4)
b/Å	23.4927(3)	19.1909(6)
c/Å	12.97151(17)	13.6635(4)
α/°	90	90
β/°	100.6831(13)	93.695(3)
γ/°	90	90
Volume/Å3	3826.57(9)	3224.84(18)
Z	4	4
ρcalcg/cm3	1.512	1.474
μ/mm-1	7.670	6.882
F(000)	1788.0	1480.0
Crystal size/mm3	0.21 × 0.09 × 0.08	0.46 × 0.22 × 0.17
Radiation	CuKα (λ = 1.54184)	CuKα (λ = 1.54184)
2Θ range for data collection/°	7.526 to 134.158	7.954 to 134.13
Index ranges	-15 ≤ h ≤ 15, -28 ≤ k ≤ 28, -15 ≤ l ≤ 15	-14 ≤ h ≤ 14, -22 ≤ k ≤ 22, -16 ≤ l ≤ 16
Reflections collected	64472	21952
Independent reflections	6840 [Rint = 0.0455, Rsigma = 0.0202]	5752 [Rint = 0.0396, Rsigma = 0.0279]
Data/restraints/paramete rs	6840/0/439	5752/0/378
Goodness-of-fit on F2	1.060	1.034
Final R indexes [I>=2σ (I)]	R1 = 0.0323, wR2 = 0.0811	R1 = 0.0414, wR2 = 0.1055
Final R indexes [all data]	R1 = 0.0360, wR2 = 0.0836	R1 = 0.0464, wR2 = 0.1095
Largest diff. peak/hole / e Å-3	1.71/-0.90	1.56/-0.90

Table S7. Bond Lengths for **Ru8.**

**Atom**	**Atom**	**Length/Å**	**Atom**	**Atom**	**Length/Å**
C(1)	N(1)	1.343(4)	C(21)	Ru(1)	1.841(3)
C(1)	N(2)	1.352(4)	C(22)	C(23)	1.405(4)
C(1)	Ru(1)	2.042(3)	C(22)	C(27)	1.400(4)
C(2)	C(3)	1.517(4)	C(23)	C(24)	1.384(4)
C(2)	N(2)	1.471(4)	C(24)	C(25)	1.387(5)
C(3)	N(1)	1.483(4)	C(25)	C(26)	1.390(4)
C(4)	C(5)	1.409(4)	C(26)	C(27)	1.386(4)
C(4)	C(9)	1.404(4)	C(27)	O(1)	1.390(3)
C(4)	N(1)	1.444(4)	C(28)	C(29)	1.510(4)
C(5)	C(6)	1.390(4)	C(28)	C(30)	1.511(4)
C(5)	C(10)	1.523(4)	C(28)	O(1)	1.465(3)
C(6)	C(7)	1.385(5)	C(31)	C(32)	1.402(4)
C(7)	C(8)	1.378(5)	C(31)	C(36)	1.404(4)
C(8)	C(9)	1.410(4)	C(31)	S(1)	1.758(3)
C(9)	C(13)	1.513(5)	C(32)	C(33)	1.386(4)
C(10)	C(11)	1.538(4)	C(32)	Cl(1)	1.743(3)
C(10)	C(12)	1.531(5)	C(33)	C(34)	1.387(5)

C(13)	C(14)	1.539(4)	C(34)	C(35)	1.387(4)
C(13)	C(15)	1.531(4)	C(35)	C(36)	1.404(4)
C(16)	C(17)	1.498(4)	C(35)	Cl(2)	1.743(3)
C(16)	N(2)	1.465(4)	C(36)	S(2)	1.750(3)
C(17)	C(18)	1.361(4)	O(1)	Ru(1)	2.258(2)
C(17)	S(3)	1.726(3)	Ru(1)	S(1)	2.2909(7)
C(18)	C(19)	1.418(5)	Ru(1)	S(2)	2.2679(7)
C(19)	C(20)	1.349(5)	C(37)	Cl(3)	1.767(4)
C(20)	S(3)	1.717(3)	C(37)	Cl(4)	1.764(4)
C(21)	C(22)	1.454(4)			

Table S8. Values of valence angles for **Ru8.**

Atom	Atom	Atom	Angle/°	Atom	Atom	Atom	Angle/°
N(1)	C(1)	N(2)	107.6(2)	C(29)	C(28)	C(30)	114.1(3)
N(1)	C(1)	Ru(1)	132.6(2)	O(1)	C(28)	C(29)	106.3(2)
N(2)	C(1)	Ru(1)	119.8(2)	O(1)	C(28)	C(30)	109.8(2)
N(2)	C(2)	C(3)	101.7(2)	C(32)	C(31)	C(36)	118.7(3)
N(1)	C(3)	C(2)	102.4(2)	C(32)	C(31)	S(1)	121.6(2)
C(5)	C(4)	N(1)	118.5(3)	C(36)	C(31)	S(1)	119.7(2)
C(9)	C(4)	C(5)	122.3(3)	C(31)	C(32)	Cl(1)	119.6(2)
C(9)	C(4)	N(1)	119.1(3)	C(33)	C(32)	C(31)	122.4(3)
C(4)	C(5)	C(10)	121.9(3)	C(33)	C(32)	Cl(1)	117.9(2)
C(6)	C(5)	C(4)	118.0(3)	C(32)	C(33)	C(34)	119.0(3)
C(6)	C(5)	C(10)	119.9(3)	C(35)	C(34)	C(33)	119.2(3)
C(7)	C(6)	C(5)	120.8(3)	C(34)	C(35)	C(36)	122.6(3)
C(8)	C(7)	C(6)	120.4(3)	C(34)	C(35)	Cl(2)	118.3(2)
C(7)	C(8)	C(9)	121.4(3)	C(36)	C(35)	Cl(2)	119.1(2)
C(4)	C(9)	C(8)	116.8(3)	C(31)	C(36)	S(2)	120.9(2)
C(4)	C(9)	C(13)	122.7(3)	C(35)	C(36)	C(31)	118.0(3)
C(8)	C(9)	C(13)	120.2(3)	C(35)	C(36)	S(2)	121.1(2)
C(5)	C(10)	C(11)	108.9(3)	C(1)	N(1)	C(3)	112.2(2)

Table S9. Values of torsion angles for **Ru8**.

A	B	C	D	Angle/	A	B	C	D	Angle/
C(2)	C(3)	N(1)	C(1)	14.3(3)	C(25)	C(26)	C(27)	C(22)	4.1(4)
C(2)	C(3)	N(1)	C(4)	-175.5(3)	C(25)	C(26)	C(27)	O(1)	-175.0(3)
C(3)	C(2)	N(2)	C(1)	16.6(3)	C(26)	C(27)	O(1)	C(28)	-1.2(4)
C(3)	C(2)	N(2)	C(16)	179.3(2)	C(26)	C(27)	O(1)	Ru(1)	-173.5(2)
C(4)	C(5)	C(6)	C(7)	1.2(5)	C(27)	C(22)	C(23)	C(24)	-0.3(4)
C(4)	C(5)	C(10)	C(11)	-101.8(3)	C(29)	C(28)	O(1)	C(27)	150.4(2)
C(4)	C(5)	C(10)	C(12)	135.8(3)	C(29)	C(28)	O(1)	Ru(1)	-38.9(3)
C(4)	C(9)	C(13)	C(14)	98.4(3)	C(30)	C(28)	O(1)	C(27)	-85.7(3)
C(4)	C(9)	C(13)	C(15)	-140.4(3)	C(30)	C(28)	O(1)	Ru(1)	84.9(3)
C(5)	C(4)	C(9)	C(8)	4.0(4)	C(31)	C(32)	C(33)	C(34)	2.8(5)
C(5)	C(4)	C(9)	C(13)	-169.9(3)	C(31)	C(36)	S(2)	Ru(1)	-0.6(2)
C(5)	C(4)	N(1)	C(1)	-104.3(3)	C(32)	C(31)	C(36)	C(35)	-0.1(4)
C(5)	C(4)	N(1)	C(3)	87.0(3)	C(32)	C(31)	C(36)	S(2)	178.6(2)
C(5)	C(6)	C(7)	C(8)	2.5(5)	C(32)	C(31)	S(1)	Ru(1)	-177.8(2)
C(6)	C(5)	C(10)	C(11)	73.2(4)	C(32)	C(33)	C(34)	C(35)	-0.8(5)

C(6)	C(5)	C(10)	C(12)	-49.2(4)	C(33)	C(34)	C(35)	C(36)	-1.6(5)
C(6)	C(7)	C(8)	C(9)	-3.1(5)	C(33)	C(34)	C(35)	Cl(2)	178.6(2)
C(7)	C(8)	C(9)	C(4)	-0.1(4)	C(34)	C(35)	C(36)	C(31)	2.1(4)
C(7)	C(8)	C(9)	C(13)	174.0(3)	C(34)	C(35)	C(36)	S(2)	-176.6(2)
C(8)	C(9)	C(13)	C(14)	-75.3(4)	C(35)	C(36)	S(2)	Ru(1)	178.0(2)
C(8)	C(9)	C(13)	C(15)	45.9(4)	C(36)	C(31)	C(32)	C(33)	-2.3(4)
C(9)	C(4)	C(5)	C(6)	-4.6(4)	C(36)	C(31)	C(32)	Cl(1)	179.1(2)
C(9)	C(4)	C(5)	C(10)	170.5(3)	C(36)	C(31)	S(1)	Ru(1)	0.9(2)
C(9)	C(4)	N(1)	C(1)	77.2(4)	Cl(1)	C(32)	C(33)	C(34)	-178.6(2)
C(9)	C(4)	N(1)	C(3)	-91.5(3)	Cl(2)	C(35)	C(36)	C(31)	-178.1(2)
C(10)	C(5)	C(6)	C(7)	-174.0(3)	Cl(2)	C(35)	C(36)	S(2)	3.2(3)
C(16)	C(17)	C(18)	C(19)	-177.4(3)	N(1)	C(1)	N(2)	C(2)	-8.3(3)
C(16)	C(17)	S(3)	C(20)	177.8(3)	N(1)	C(1)	N(2)	C(16)	-169.4(3)
C(17)	C(16)	N(2)	C(1)	-136.4(3)	N(1)	C(4)	C(5)	C(6)	176.9(3)
C(17)	C(16)	N(2)	C(2)	63.4(3)	N(1)	C(4)	C(5)	C(10)	-7.9(4)
C(17)	C(18)	C(19)	C(20)	-0.2(4)	N(1)	C(4)	C(9)	C(8)	-177.5(3)
C(18)	C(17)	S(3)	C(20)	0.5(2)	N(1)	C(4)	C(9)	C(13)	8.5(4)
C(18)	C(19)	C(20)	S(3)	0.6(4)	N(2)	C(1)	N(1)	C(3)	-4.4(3)
C(19)	C(20)	S(3)	C(17)	-0.7(3)	N(2)	C(1)	N(1)	C(4)	-173.8(3)
C(21)	C(22)	C(23)	C(24)	179.7(3)	N(2)	C(2)	C(3)	N(1)	-17.2(3)
C(21)	C(22)	C(27)	C(26)	177.0(3)	N(2)	C(16)	C(17)	C(18)	-107.4(3)
C(21)	C(22)	C(27)	O(1)	-3.9(4)	N(2)	C(16)	C(17)	S(3)	75.8(3)
C(22)	C(21)	Ru(1)	C(1)	99.8(2)	Ru(1)	C(1)	N(1)	C(3)	174.2(2)
C(22)	C(21)	Ru(1)	O(1)	5.2(2)	Ru(1)	C(1)	N(1)	C(4)	4.7(4)
C(22)	C(21)	Ru(1)	S(1)	-80.0(2)	Ru(1)	C(1)	N(2)	C(2)	172.93(19)
C(22)	C(21)	Ru(1)	S(2)	-170.5(2)	Ru(1)	C(1)	N(2)	C(16)	11.8(4)
C(22)	C(23)	C(24)	C(25)	2.5(5)	Ru(1)	C(21)	C(22)	C(23)	177.2(2)
C(22)	C(27)	O(1)	C(28)	179.7(2)	Ru(1)	C(21)	C(22)	C(27)	-2.8(4)
C(22)	C(27)	O(1)	Ru(1)	7.4(3)	S(1)	C(31)	C(32)	C(33)	176.4(2)
C(23)	C(22)	C(27)	C(26)	-3.1(4)	S(1)	C(31)	C(32)	Cl(1)	-2.2(3)
C(23)	C(22)	C(27)	O(1)	176.1(2)	S(1)	C(31)	C(36)	C(35)	-178.9(2)
C(23)	C(24)	C(25)	C(26)	-1.6(5)	S(1)	C(31)	C(36)	S(2)	-0.2(3)
C(24)	C(25)	C(26)	C(27)	-1.7(5)	S(3)	C(17)	C(18)	C(19)	-0.3(4)

Table S10. Bond Lengths for **Ru10**.

Atom	Atom	Length/Å	Atom	Atom	Length/Å
C1	C4	1.542(5)	C20	C21	1.403(6)
C1	N1	1.322(5)	C20	C25	1.400(6)
C1	Ru1	2.007(4)	C21	C22	1.391(6)
C2	C3	1.530(5)	C21	O1	1.395(5)
C2	C15	1.529(5)	C22	C23	1.383(6)
C2	C16	1.520(5)	C23	C24	1.388(7)
C2	N1	1.538(4)	C24	C25	1.382(6)
C3	C4	1.530(5)	C26	C27	1.495(6)
C4	C17	1.539(5)	C26	C28	1.519(6)

C4	C18	1.541(5)	C26	O1	1.479(5)
C5	C6	1.410(5)	C29	C30	1.401(5)
C5	C10	1.412(5)	C29	C34	1.413(5)
C5	N1	1.460(5)	C29	S1	1.755(4)
C6	C7	1.400(6)	C30	C31	1.413(5)
C6	C11	1.515(5)	C30	S2	1.743(4)
C7	C8	1.383(6)	C31	C32	1.386(6)
C8	C9	1.375(5)	C31	Cl1	1.737(4)
C9	C10	1.393(5)	C32	C33	1.383(6)
C10	C13	1.502(5)	C33	C34	1.366(6)
C11	C12	1.505(5)	C34	Cl2	1.741(4)
C13	C14	1.527(5)	O1	Ru1	2.298(3)
C19	C20	1.449(5)	Ru1	S1	2.3326(9)
C19	Ru1	1.838(4)	Ru1	S2	2.2843(9)

Table S11. Values of valence angles for **Ru10**.

Atom	Atom	Atom	Angle/°	Atom	Atom	Atom	Angle/°
C4	C1	Ru1	118.6(2)	C22	C23	C24	121.0(4)
N1	C1	C4	107.7(3)	C25	C24	C23	120.0(4)
N1	C1	Ru1	133.1(3)	C24	C25	C20	120.5(4)
C3	C2	N1	99.5(3)	C27	C26	C28	112.9(4)
C15	C2	C3	112.1(3)	O1	C26	C27	108.4(3)
C15	C2	N1	111.8(3)	O1	C26	C28	109.5(3)
C16	C2	C3	113.5(3)	C30	C29	C34	117.6(4)
C16	C2	C15	108.1(3)	C30	C29	S1	119.7(3)
C16	C2	N1	111.7(3)	C34	C29	S1	122.5(3)
C4	C3	C2	106.6(3)	C29	C30	C31	118.8(3)
C3	C4	C1	102.4(3)	C29	C30	S2	121.1(3)
C3	C4	C17	111.4(3)	C31	C30	S2	120.1(3)
C3	C4	C18	112.3(3)	C30	C31	Cl1	119.5(3)
C17	C4	C1	115.4(3)	C32	C31	C30	121.9(4)
C17	C4	C18	107.0(3)	C32	C31	Cl1	118.6(3)
C18	C4	C1	108.4(3)	C33	C32	C31	118.9(4)
C6	C5	C10	121.9(3)	C34	C33	C32	120.1(4)
C6	C5	N1	118.4(3)	C29	C34	Cl2	119.3(3)
C10	C5	N1	119.7(3)	C33	C34	C29	122.6(4)
C5	C6	C11	121.3(3)	C33	C34	Cl2	118.0(3)
C7	C6	C5	117.4(4)	C1	N1	C2	115.3(3)
C7	C6	C11	121.1(3)	C1	N1	C5	125.5(3)
C8	C7	C6	121.2(4)	C5	N1	C2	119.2(3)
C9	C8	C7	120.2(4)	C21	O1	C26	116.8(3)
C8	C9	C10	121.6(4)	C21	O1	Ru1	109.5(2)
C5	C10	C13	123.6(3)	C26	O1	Ru1	133.7(2)
C9	C10	C5	117.6(3)	C1	Ru1	O1	103.35(12)

C9	C10	C13	118.6(3)	C1	Ru1	S1	144.39(10)
C12	C11	C6	116.2(4)	C1	Ru1	S2	83.82(10)
C10	C13	C14	114.7(3)	C19	Ru1	C1	98.89(15)
C20	C19	Ru1	120.0(3)	C19	Ru1	O1	78.70(13)
C21	C20	C19	118.6(3)	C19	Ru1	S1	116.23(11)
C25	C20	C19	123.1(4)	C19	Ru1	S2	95.57(12)
C25	C20	C21	118.3(4)	O1	Ru1	S1	89.74(7)
C22	C21	C20	121.4(4)	S2	Ru1	O1	171.35(7)
C22	C21	O1	125.4(4)	S2	Ru1	S1	87.00(3)
O1	C21	C20	113.2(3)	C29	S1	Ru1	105.07(13)
C23	C22	C21	118.8(4)	C30	S2	Ru1	105.75(13)

Table S12. Values of torsion angles for **Ru10**.

A	B	C	D	Angle/°	A	B	C	D	Angle/°
C2	C3	C4	C1	29.4(4)	C22	C23	C24	C25	0.0(7)
C2	C3	C4	C17	153.3(3)	C23	C24	C25	C20	-1.0(7)
C2	C3	C4	C18	-86.6(4)	C25	C20	C21	C22	1.8(6)
C3	C2	N1	C1	12.5(4)	C25	C20	C21	O1	-178.8(3)
C3	C2	N1	C5	-169.5(3)	C27	C26	O1	C21	158.7(4)
C4	C1	N1	C2	5.8(4)	C27	C26	O1	Ru1	-22.8(5)
C4	C1	N1	C5	-172.1(3)	C28	C26	O1	C21	-77.8(4)
C5	C6	C7	C8	1.3(6)	C28	C26	O1	Ru1	100.7(4)
C5	C6	C11	C12	-173.4(4)	C29	C30	C31	C32	1.3(6)
C5	C10	C13	C14	-134.2(4)	C29	C30	C31	Cl1	-177.9(3)
C6	C5	C10	C9	2.5(5)	C29	C30	S2	Ru1	12.8(3)
C6	C5	C10	C13	-171.9(3)	C30	C29	C34	C33	3.3(6)
C6	C5	N1	C1	83.9(4)	C30	C29	C34	Cl2	-177.7(3)
C6	C5	N1	C2	-93.9(4)	C30	C29	S1	Ru1	-1.3(3)
C6	C7	C8	C9	1.9(6)	C30	C31	C32	C33	1.9(6)
C7	C6	C11	C12	1.3(6)	C31	C30	S2	Ru1	-167.2(3)
C7	C8	C9	C10	-3.1(6)	C31	C32	C33	C34	-2.4(6)
C8	C9	C10	C5	0.9(6)	C32	C33	C34	C29	-0.2(6)
C8	C9	C10	C13	175.5(4)	C32	C33	C34	Cl2	-179.2(3)
C9	C10	C13	C14	51.5(5)	C34	C29	C30	C31	-3.7(5)
C10	C5	C6	C7	-3.5(5)	C34	C29	C30	S2	176.3(3)
C10	C5	C6	C11	171.4(3)	C34	C29	S1	Ru1	174.3(3)
C10	C5	N1	C1	-96.2(4)	Cl1	C31	C32	C33	-178.9(3)
C10	C5	N1	C2	86.0(4)	N1	C1	C4	C3	-21.6(4)
C11	C6	C7	C8	-173.6(4)	N1	C1	C4	C17	-142.8(3)
C15	C2	C3	C4	-143.8(3)	N1	C1	C4	C18	97.2(4)
C15	C2	N1	C1	131.1(3)	N1	C2	C3	C4	-25.4(4)
C15	C2	N1	C5	-51.0(4)	N1	C5	C6	C7	176.4(3)
C16	C2	C3	C4	93.3(4)	N1	C5	C6	C11	-8.7(5)
C16	C2	N1	C1	-107.6(4)	N1	C5	C10	C9	-177.5(3)

C16	C2	N1	C5	70.3(4)	N1	C5	C10	C13	8.2(5)
C19	C20	C21	C22	-179.1(4)	O1	C21	C22	C23	177.9(4)
C19	C20	C21	O1	0.4(5)	Ru1	C1	C4	C3	150.3(2)
C19	C20	C25	C24	-179.0(4)	Ru1	C1	C4	C17	29.1(4)
C20	C19	Ru1	C1	-103.2(3)	Ru1	C1	C4	C18	-90.9(3)
C20	C19	Ru1	O1	-1.3(3)	Ru1	C1	N1	C2	-164.5(3)
C20	C19	Ru1	S1	82.8(3)	Ru1	C1	N1	C5	17.7(5)
C20	C19	Ru1	S2	172.2(3)	Ru1	C19	C20	C21	1.0(5)
C20	C21	C22	C23	-2.7(7)	Ru1	C19	C20	C25	-179.9(3)
C20	C21	O1	C26	177.6(3)	S1	C29	C30	C31	172.1(3)
C20	C21	O1	Ru1	-1.3(4)	S1	C29	C30	S2	-7.9(4)
C21	C20	C25	C24	0.1(6)	S1	C29	C34	C33	-172.4(3)
C21	C22	C23	C24	1.7(7)	S1	C29	C34	Cl2	6.6(5)
C22	C21	O1	C26	-2.9(6)	S2	C30	C31	C32	-178.7(3)
C22	C21	O1	Ru1	178.2(4)	S2	C30	C31	Cl1	2.1(4)

References

1. Müller, D. S., Curbet, I., Raoul, Y., Le Nôtre, J., Baslé, O., and Mauduit, M., Stereoretentive Olefin Metathesis Made Easy: In Situ Generation of Highly Selective Ruthenium Catalysts from Commercial Starting Materials. *Org. Lett.* **2018**, 20, 21, 6822-6826.

2. Ahmed, T. S.; Grubbs, R. H., A Highly Efficient Synthesis of Z-Macrocycles Using Stereoretentive, Ruthenium-Based Metathesis Catalysts. *Angew. Chem. Int. Ed.* **2017,** *56* (37), 11213-11216.
